# Graph regularized non-negative matrix factorization with $L_{2,1}$ norm regularization terms for drug–target interactions prediction

**DOI:** 10.1186/s12859-023-05496-6

**Published:** 2023-10-03

**Authors:** Junjun Zhang, Minzhu Xie

**Affiliations:** 1 Key Laboratory of Computing and Stochastic Mathematics(LCSM) (Ministry of Education), School of Mathematics and Statistics, Hunan Normal University, Changsha, 410081 China; 2 College of Information Science and Engineering, Hunan Normal University, Changsha, 410081 China

**Keywords:** Drug–target interactions, $L_{2,1}$ norm, Inertial proximal alternating linearized minimization

## Abstract

**Background:**

Identifying drug–target interactions (DTIs) plays a key role in drug development. Traditional wet experiments to identify DTIs are costly and time consuming. Effective computational methods to predict DTIs are useful to speed up the process of drug discovery. A variety of non-negativity matrix factorization based methods are proposed to predict DTIs, but most of them overlooked the sparsity of feature matrices and the convergence of adopted matrix factorization algorithms, therefore their performances can be further improved.

**Results:**

In order to predict DTIs more accurately, we propose a novel method iPALM-DLMF. iPALM-DLMF models DTIs prediction as a problem of non-negative matrix factorization with graph dual regularization terms and $L_{2,1}$ norm regularization terms. The graph dual regularization terms are used to integrate the information from the drug similarity matrix and the target similarity matrix, and $L_{2,1}$ norm regularization terms are used to ensure the sparsity of the feature matrices obtained by non-negative matrix factorization. To solve the model, iPALM-DLMF adopts non-negative double singular value decomposition to initialize the nonnegative matrix factorization, and an inertial Proximal Alternating Linearized Minimization iterating process, which has been proved to converge to a KKT point, to obtain the final result of the matrix factorization. Extensive experimental results show that iPALM-DLMF has better performance than other state-of-the-art methods. In case studies, in 50 highest-scoring proteins targeted by the drug gabapentin predicted by iPALM-DLMF, 46 have been validated, and in 50 highest-scoring drugs targeting prostaglandin-endoperoxide synthase 2 predicted by iPALM-DLMF, 47 have been validated.

**Supplementary Information:**

The online version contains supplementary material available at 10.1186/s12859-023-05496-6.

## Background

Determining the drug–target interactions (DTIs) is a key step in drug development process [1]. However, identifying the DTIs via wet experiments is time consuming and expensive [2, 3]. To reduce the consumption of expensive wet experiments, a variety of computational prediction models for DTIs have been proposed. The existing models for DTIs prediction mainly fall into two categories [4]. The first category formulates the interaction prediction as a binary classification task [5]. The second category aims to estimate the interaction strength of drug–target pairs [6, 7]. This paper focuses on the first category. The first category of DTI prediction models could be further grouped into ligand-based models, docking simulation based models, and chemogenomics based models [8].

Ligand-based models assume that similar ligands would interact with similar proteins [9].

The ligand based models require that a certain number of binding ligands of a given protein target should be known [10]. Docking simulation based models are based on crystal structures of target binding sites and docking simulations [11]. However, obtaining the crystal structure of a target binding site is challenging. Therefore, docking simulation based models couldn’t apply to large scale DTIs prediction.

To avoid above difficulties, chemogenomics based models use known target-drug interactions, chemical structures of drugs, genomic sequences of target proteins, and/or other related information of targets and drugs to predict potential target-drug interactions. The chemogenomics based models [8] usually use a DTI network to present the known drug–target interactions, and adopt machine learning or deep learning to predict DTIs. For example, based on the DTI network, Yamanishi et al. [12] proposed a bipartite graph learning method to predict DTIs by mapping the chemical structure space of drugs and the genomic sequence space of proteins into a unified space. In order to predict target proteins for a given drug, and the drugs targeting a given protein, Bleakley and Yamanishi [13] proposed bipartite local models (BLM), which transformed edge-prediction problems into binary classification problems. RLS-WNN [14], BLM-NII [15] and WKNKN [16] were proposed by integrating the neighbor information of similarity networks of drugs and targets.

In addition to chemical structures of drugs and genomic sequences of target proteins, some works have incorporated multiple types of information, such as side-effects [17, 18], protein-protein interactions [19], drug-disease associations [20], protein-disease associations [21] and gene ontology information [22] for DTIs prediction. In order to integrate multiple types of information, random walk with restart (RWR) [23, 24] was used to capture topological relations between nodes in the heterogeneous network. In addition, 2D structural images of drugs [25] and 3D structures of the proteins [26] were also used as input data for DTIs prediction.

As a kind of machine learning method, matrix factorization has also been used to predict DTIs and has achieved better performance than other machine learning methods [2]. In DTIs prediction, a DTI matrix is usually used to represent the known drug–target interactions. Matrix factorization decomposes the interaction matrix into two low rank matrices, which represent the feature matrices of drugs and targets. The optimization object of matrix factorization based DTIs prediction methods is that the product of the feature matrices of drugs and targets approximates the interaction matrix of drugs and targets as closely as possible. For example, Gönen [27] proposed a kernelized Bayesian matrix factorization with twin kernels method to predict DTIs. Bolgár and Antal [28] proposed a fusion method, called a variational Bayesian multiple kernel logistic matrix factorization method, which used graph Laplacian regularization, multiple kernel learning, and a variational Bayesian inference process to infer interactions. In order to learn the values of missing entries in DTI matrix, a variety of methods with regularization terms were proposed based on matrix factorization, such as MSCMF [29], NRLMF [30], GRMF [31], $L_{2,1}$-GRMF [32] and SRCMF [33]. Recently, Ding et al. [34] proposed a multiple kernel-based triple collaborative matrix factorization (MK-TCMF) method. MK-TCMF used Multi-kernel learning (MKL) to integrate different similarities of drugs and targets, and used triple collaborative matrix factorization to decompose the original DTI matrix into three matrices: a latent feature matrix of drugs, latent feature matrix of targets and a bi-projection matrix.

To solve matrix factorization problems, the above methods used either the alternating least squares algorithm [35] or the multiplicative update algorithm [36]. However, it is difficult to guarantee that the above algorithms converge to a stationary point [37]. Recently, Pock and Sabach [38] proposed an inertial version of the Proximal Alternating Linearized Minimization algorithm (iPALM), which can be used to solve non-negative matrix factorization, and iPALM has been proven to converge to a stationary point.

In this paper, we propose a novel method iPALM-DLMF. iPALM-DLMF models DTIs prediction as a problem of non-negative matrix factorization with graph dual regularization terms and $L_{2,1}$ norm regularization terms. The graph dual regularization terms are used to integrate the information from the drug similarity matrix and the target similarity matrix, and $L_{2,1}$ norm regularization terms are used to ensure the sparsity of the matrices obtained by non-negative matrix factorization. To solve the model, non-negative double singular value decomposition (NNDSVD) [39] is used to initialize the nonnegative matrix factorization, and an inertial Proximal Alternating Linearized Minimization iterating process is used to obtain the final matrix factorization.

The main contributions of iPALM-DLMF are as follows:

1. Improving the non-negative matrix factorization model by adding graph dual regularization terms and $L_{2,1}$ norm regularization terms.
2. $L_{2,1}$ norm regularization terms ensure sparsity of the matrices obtained by non-negative matrix factorization.
3. The inertial proximal alternating linearized minimization algorithm with fast convergence is used to solve the matrix factorization.

Extensive experimental results show that iPALM-DLMF has better performance than other state-of-the-art methods. In case studies involving the drug gabapentin and the target prostaglandin-endoperoxide synthase 2, 46 of the 50 highest-scoring highest-scoring targets predicted to interact with gabapentin and 47 of the 50 highest-scoring drugs predicted to interact with prostaglandin-endoperoxide synthase 2 have been validated by wet experiments. The case studies show that, for drugs that do not have any known target proteins and for proteins that are so far not approved as drug targets, iPALM-DLMF also has good prediction performance.

## Materials

In order to evaluate prediction performance of the proposed iPALM-DLMF, we used the same four benchmark datasets as used by most similar works. The information of the four datasets are shown in Table 1. Each dataset contains three types of information: known drug–target interactions, drug chemical structures and target protein sequences. The datasets correspond to different target protein types, including nuclear receptors (NR), G protein-coupled receptors (GPCR), ion channels (IC) and enzymes (E). Accordingly, the four datasets are called NR, GPCR, IC and E. The four datasets were built by Yamanishi et al. [12] from public databases BRENDA [40], KEGG BRITE [41], SuperTarget [42] and DrugBank [43], and are publicly available at http://web.kuicr.kyoto-u.ac.jp/supp/yoshi/drugtarget/. The known interactions between *n* drugs and *m* proteins are recorded by a $n \times m$ DTI matrix *Z*. If the *i*th drug is approved to target the *j*th protein, $Z_{i,j}=1$; otherwise $Z_{i,j}=0$.

The structural similarities between drugs are calculated using SIMCOMP [44] according to the size of the common substructures between two drugs. The similarity information of *n* drugs are stored in a $n\times n$ matrix $S^d$.

The normalized version of the Smith-Waterman score is used to calculate the sequence similarity of the target proteins [45]. Let $p_1$ and $p_2$ represent two proteins. The Smith-Waterman score of the standardized version of $p_1$ and $p_2$ is $s({p_1},{p_2}) = \frac{{SW({p_1},{p_2})}}{{\sqrt{SW({p_1},{p_1})} \sqrt{SW({p_2},{p_2})} }}$, where *SW*(., .) be the original Smith-Waterman alignment score. The similarity information of *m* target proteins are denoted by a $m\times m$ matrix $S^t$.

**Table 1:** The information of the benchmark datasets

Datasets	NR	GPCR	IC	E
Interactions	90	635	1476	2926
Drugs	54	223	210	445
Targets	26	95	204	664
Sparsity(%)	93.59	97.00	96.55	99.01

## Methods

**Fig. 1:**
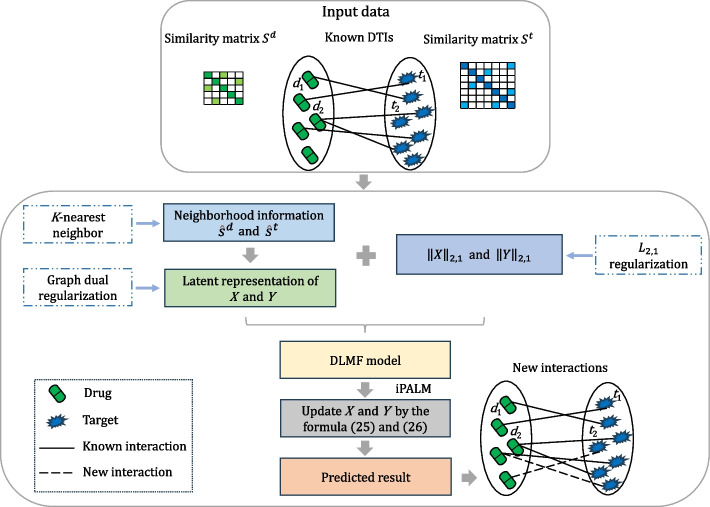
A brief flow chart of iPALM-DLMF

iPALM-DLMF models DTIs prediction problem as a non-negative factorization problem with graph dual regularization terms and $L_{2,1}$ norm regularization terms. iPALM-DLMF takes the DTI matrix *Z*, drug similarity matrix $S^d$ and target similarity matrix $S^t$ as inputs, uses $S^d$ and $S^t$ to construct graph dual regularization terms, and solve non-negative matrix factorization problem of *Z* with graph dual regularization terms and $L_{2,1}$ norm regularization terms to obtain the feature matrices of drugs and targets. Finally the feature matrices are utilized to predict DTIs. A brief flow chart of iPALM-DLMF is shown in Fig. 1.

### Non-negative matrix factorization

In DTIs prediction, the non-negativity matrix factorization (NMF) of the DTI matrix is widely used to obtain low-dimensional feature representations of drugs and targets in the DTI space. The general form of the NMF is as follows:

1 $$\begin{aligned}{} & {} \min \left\| {Z - X{Y^T}} \right\| _F^2\nonumber \\{} & {} s.t. ~ X \ge 0, Y \ge 0. \end{aligned}$$

where *X* and *Y* represent the latent feature matrices of drugs and targets, respectively. *k* is the rank of *X* and *Y*, $k \ll \min (m,n)$, $X \in {\mathbb {R}^{n \times k}}, Y \in {\mathbb {R}^{m \times k}}$. The non-negativity constraint terms are adopted to ensure non-negativity of *X* and *Y*.

### Graph dual regularized non-negative matrix factorization

As a embedding model, the learning performance of NMF can be greatly improved if the geometrical information has been taken into account [46]. Cai et al. [47] used a graph regularization item to integrate the geometric information. Furthermore, Shang et al. [48] introduced graph dual regularization items based on both data manifold and feature manifold.

In order to obtain geometric information of drugs and targets, two *K*-nearest neighbor graphs $N^d$ and $N^t$ of drugs and targets respectively are constructed based on $S^d$ and $S^t$, respectively.

For two drugs $d_i$ and $d_j$, the weight of the edge between vertices *i* and *j* in graph $N^d$ is defined as follows.

2 $$\begin{aligned} {N_{ij}^d} = \left\{ \begin{array}{l} 1,j \in {\mathcal {N}_K}(i) \text { and } i \in {\mathcal {N}_K}(j) \\ 0,j \notin {\mathcal {N}_K}(i) \text { and } i \notin {\mathcal {N}_K}(j) \\ 0.5, \text {otherwise,} \\ \end{array} \right. \end{aligned}$$

where $\mathcal {N}_K(i)$ denotes the sets of *K* most similar drugs of drugs $d_i$ according to $S^d$. Based on $N^d$ and $S^d$, a sparse matrix ${\hat{S}}_{ij}^d$ is computed as follows.

3 $$\begin{aligned} {\hat{S}}_{ij}^d = {N_{ij}^d}S_{ij}^d, \forall i, j. \end{aligned}$$

${\hat{S}}^d$ is a weight matrix representing the drug neighbor graph. The graph Laplacian of ${\hat{S}}^d$ is ${\mathcal {L}_d} = {D^d} - {{\hat{S}}^d}$, where $D^d$ is a diagonal degree matrix with $D_{ii}^d = \sum \limits _r {{\hat{S}}_{ir}^d}$.

Similarly, the weight matrix ${\hat{S}}^t$ corresponding to the target neighbor graph is computed as follows.

4 $$\begin{aligned} {\hat{S}}_{ij}^t = {N_{ij}^t}S_{ij}^t, \forall i, j. \end{aligned}$$

The graph Laplacian of ${\hat{S}}^t$ is ${\mathcal {L}_t} = {D^t} - {{\hat{S}}^t}$, where $D^t$ is diagonal degree matrix with $D_{jj}^t = \sum \limits _q {{\hat{S}}_{jq}^t}$.

The normalized graph Laplacian forms of $\mathcal {L}_d$ and $\mathcal {L}_t$ are as follows.

5 $$\begin{aligned} {\widetilde{{\mathcal {L}}}_d}= & {} {\left( {{D^d}} \right) ^{ - 1/2}}{{{\mathcal {L}}}_d}{\left( {{D^d}} \right) ^{ - 1/2}}, \end{aligned}$$

6 $$\begin{aligned} {\widetilde{{\mathcal {L}}}_t}= & {} {\left( {{D^t}} \right) ^{ - 1/2}}{{{\mathcal {L}}}_t}{\left( {{D^t}} \right) ^{ - 1/2}}. \end{aligned}$$

The optimization model of graph dual regularization non-negative matrix factorization (GDNMF) of the drug-protein interaction matrix *Z* is formulated as follows.

7 $$\begin{aligned}{} & {} \mathop {\min }\limits _{(X,Y)}\frac{1}{2}\left\| {Z - X{Y^T}} \right\| _F^2 +{\lambda _d}{\text {Tr}}({X^T}\widetilde{\mathcal {L}}_d X)\nonumber \\{} & {} \quad + {\lambda _t}{\text {Tr}}({Y^T}\widetilde{\mathcal {L}}_t Y).\nonumber \\{} & {} s.t. X \ge 0, Y \ge 0, \end{aligned}$$

where $\lambda _d$ and $\lambda _t$ are regularization parameters.

### GDNMF with $L_{2,1}$-norm regularization terms

In order to ensure sparsity of the matrices obtained by non-negative matrix factorization, we introduce the $L_{2,1}$-norm of *X* and *Y* into GDNMF optimization model, and the optimization model of GDNMF with $L_{2,1}$-norm regularization terms is formatted as follows.

8 $$\begin{aligned}{} & {} \mathop {\min }\limits _{(X,Y)}\frac{1}{2}\left\| {Z - X{Y^T}} \right\| _F^2 +{\lambda _d}{\text {Tr}}({X^T}\widetilde{\mathcal {L}}_d X)\nonumber \\{} & {} \quad + {\lambda _t}{\text {Tr}}({Y^T}\widetilde{\mathcal {L}}_t Y) + {\lambda _l}(\left\| X \right\| _{2,1} + \left\| Y \right\| _{2,1}),\nonumber \\{} & {} \quad s.t. ~ X \ge 0, Y \ge 0, \end{aligned}$$

where $\lambda _l$ is a regularization parameter, ${\left\| X \right\| _{2,1}}$ and ${\left\| Y \right\| _{2,1}}$ represent $L_{2,1}$ norms of matrix *X* and *Y*, respectively, and ${\left\| X \right\| _{2,1}} = {\sum \limits _i {({{\sum \limits _j {({x_{ij}})} }^2})} ^{1/2}}$, ${\left\| Y \right\| _{2,1}} = {\sum \limits _i {({{\sum \limits _j {({y_{ij}})} }^2})} ^{1/2}}$.

### Algorithm

#### Non-negative double singular value decomposition

To provide better and explainable initial component matrices for matrix factorization, non-negative double singular value decomposition (NNDSVD) [39] is adopted to obtain initial value of matrix factorization. NNDSVD is an algorithm based on SVD of *Z*. $Z = \sum _{i = 1,.., k}{\sigma _i u_i v_i^T}$, where *Z* equals to the sum of *k* leading singular factors, $u_i$ and $v_i$ denote the left and right singular vectors corresponding to $\sigma _i$, respectively, and $\sigma$ denotes singular value of *Z*.

For a vector or matrix *z*, $z^+=max(0,z)$ represents nonnegative section of *z*, $z^-=max(0,-z)$ represents nonpositive section of *z*, $z=z^+-z^-$. $Z = \sum _{i = 1,.., k}{\sigma _i u_i v_i^T}$ can be transformed to the following form:

9 $$\begin{aligned} Z= & {} \sum _{i = 1,.., k}{u_i v_i} \nonumber \\= & {} \sum _{i = 1,.., k}{(u_{i}^+ v_{i}^+ + u_{i}^- v_{i}^-)- (u_{i}^- v_{i}^+ + u_{i}^+ v_{i}^-)}. \end{aligned}$$

If $\left\| {u_i^ + } \right\| \left\| {v_i^ + } \right\| > \left\| {u_i^ - } \right\| \left\| {v_i^ - } \right\|$, $\sqrt{\sigma _i ||u^+_{i} || ||v^+_{i}|| } ( u^+_{i} / ||u^+_{i} ||)$ is used to obtain initial value of *i*-th column of *X*. $\sqrt{\sigma _i\left\| u_i^{+}\right\| \left\| v_i^{+}\right\| }\left( v_i^{+} /\left\| v_i^{+}\right\| \right)$ is used to obtain initial value of *i*-th column of *Y*. Otherwise, $\sqrt{\sigma _i\left\| u_i^{-}\right\| \left\| v_i^{-}\right\| }$ $\left( u_i^{-} /\left\| u_i^{-}\right\| \right)$ and $\sqrt{\sigma _i\left\| u_i^{-}\right\| \left\| v_i^{-}\right\| }\left( v_i^{-} /\left\| v_i^{-}\right\| \right)$. The detailed steps of NNDSVD are shown in the Additional file 1: Table S1, 2.

####  Proximal alternating linearized minimization

Bolte et al. [49] proposed a Proximal Alternating Linearized Minimization method (PALM), which can be regarded as a blockwise application of the proximal forward-backward algorithm [50, 51] in the nonconvex setting.

Model (8) can be transformed to the following form:

10 $$\begin{aligned}{} & {} \mathop {\min }\limits _{(X,Y)}\frac{1}{2}\left\| {Z - X{Y^T}} \right\| _F^2+R(X)+R(Y)\nonumber \\{} & {} \quad s.t. ~ X \ge 0, Y \ge 0, \end{aligned}$$

where $R(X)={\lambda _d}{\text {Tr}}({X^T}\widetilde{\mathcal {L}}_d X)+{\lambda _l}\left\| X \right\| _{2,1}$, $R(Y)={\lambda _t}{\text {Tr}}({Y^T}\widetilde{\mathcal {L}}_t Y) +{\lambda _l} \left\| Y \right\| _{2,1}$. The non-negative constraint of formula (10) can be transformed to the following form:

11 $$\begin{aligned} X \ge 0 \rightarrow {\delta _X}= & {} \left\{ \begin{array}{l} X,X \ge 0,\\ \infty ,otherwise, \\ \end{array} \right. \end{aligned}$$

12 $$\begin{aligned} Y \ge 0 \rightarrow {\delta _Y}= & {} \left\{ \begin{array}{l} Y,Y \ge 0, \\ \infty ,otherwise.\\ \end{array} \right. \end{aligned}$$

Then the model (10) is transformed into the following form:

13 $$\begin{aligned} \mathop {\min }\psi (X,Y)= & {} \mathop {\min }\frac{1}{2}\left\| {Z - X{Y^T}} \right\| _F^2\nonumber \\{} & {} +R(X)+R(Y)+ \delta _X+ \delta _Y. \end{aligned}$$

Gauss-Seidel method is adopted to solve model (13). The schemes are as follows,

14 $$\begin{aligned}{} & {} {X^{i + 1}} \in \arg \min _{X} \psi (X,{Y^i}), \end{aligned}$$

15 $$\begin{aligned}{} & {} {Y^{i + 1}} \in \arg \min _{Y} \psi ({X^{i + 1}},Y). \end{aligned}$$

Let $G(X,Y)=\frac{1}{2}\left\| {Z - X{Y^T}} \right\| _F^2+R(X)+R(Y)$. We remove the constant terms by plugging $Y^i$ into $\psi (X,Y)$ and get ${X^{i + 1}} \in \arg \min \{ {\delta _X} +R(X)+ \left\| {Z - X{Y^T}} \right\| _F^2\}$, where $G(X,Y^i)$ is smooth function. After removing the constant term, the second-order Taylor series of $G(X,Y^i)$ at a point $X^i$ is given by:

16 $$\begin{aligned}{} & {} {X^{i + 1}} \in \arg \min _{X} \{ \left\langle {X - {X^i},{\nabla _X}G({X^i},{Y^i})} \right\rangle \nonumber \\{} & {} \quad + \frac{1}{2}{\nabla _X}({\nabla _X}G({X^i},{Y^i})){\left\| {X - {X^i}} \right\| _{F}^2} + {\delta _X}\}, \end{aligned}$$

where ${\nabla _X}G$ is the partial derivative of *G* with respect to *X*.

Define the proximal map of *f*: $prox_t^{f} = \arg \min \{ f (u)$ $+ \frac{1}{2t}{\left\| {u - x} \right\| _{F}^2},u \in \mathbb {R}{^d}\}$, where $f: \mathbb {R}{^d} \rightarrow ( - \infty , + \infty ]$ is the lower semi-continuous function to ensure non-negativity, *x* is a fixed point, *t* is a constant, $x\in \mathbb {R}{^d}$, $t>0$. According to the definition of proximal map, the solution of formula (16) is as follows (the detailed derivation processes are shown in Appendix):

17 $$\begin{aligned} {X^{i + 1}} \in prox_{{c _1^i}}^{\delta _X}({X^i} - \frac{1}{{{c _1^i}}}{\nabla _X}G({X^i},{Y^i})). \end{aligned}$$

Similarity, $${Y^{i + 1}} \in prox_{c_2^i}^{\delta _Y}({Y^i} - \frac{1}{{c_2^i}}{\nabla _Y}G({X^{i + 1}},{Y^i}))$$, where $$\left\{ \begin{array}{l} c_1^i = {\nabla _X}({\nabla _X}G({X^i},{Y^i})) = {\left\| {{Y^i}{{({Y^i})}^T}} \right\| _F}, \\ c_2^i = {\nabla _Y}({\nabla _Y}G({X^i},{Y^i})) = {\left\| {{X^i}{{({X^i})}^T}} \right\| _F}. \\ \end{array} \right.$$

Let

18 $$\begin{aligned} U^i={X^i} - \frac{1}{{{c _1^i}}}{\nabla _X}G({X^i},{Y^i}). \end{aligned}$$

The formula (17) is translated to

19 $$\begin{aligned} {X^{i + 1}} \in prox_{{c _1^i}}^{\delta _X}U^i= \max \{ 0,U\}, \end{aligned}$$

where $prox_{{c _1^i}}^{\delta _X}U^i$ is a map, which project on $\mathbb {R}_{+}^{m\times n}$. Similarity, we have

20 $$\begin{aligned} {Y^{i + 1}}= & {} \mathop {\arg \min }\limits _Y \psi (X^i,Y)\nonumber \\= & {} \max \{ 0,{{Y^i} - }{\frac{1}{{c_2^i}}{\nabla _Y}G({X^{i + 1}},{Y^i})}\}. \end{aligned}$$

For a sequence ${(X^i, Y^i)}_{i \in \mathbb {N}}$, parameters $c _1^i$ and $c_2^i$, we can get

21 $$\begin{aligned} \left\{ \begin{array}{c} {X^{i + 1}} \in prox_{c _1^i}^{\delta _X}({X^i} - \frac{1}{{c _1^i}}{\nabla _X}G({X^i},{Y^i})), \\ {Y^{i + 1}} \in prox_{c_2^i}^{\delta _Y}({Y^i} - \frac{1}{{c_2^i}}{\nabla _Y}G({X^{i + 1}},{Y^i})), \\ \end{array} \right. \end{aligned}$$

#### Inertial terms

Alvarez and Attouch [52] first proposed the ideal of inertia in 2001, which was applied in an proximal method for maximal monotone operators via discretization of a nonlinear oscillator with damping. Polyak showed that inertial terms can speed up convergence for the standard gradient method, while the cost of each iteration stays basically unchanged [53, 54]. In PALM, the optimization scheme is an first-order gradient descent method. In order to accelerate the PALM, inertial terms are used.

#### Inertial proximal alternating linearized minimization

We uses *G* to denote the object function of the model (8), *i.e. *

22 $$\begin{aligned} G(X, Y)= & {} \frac{1}{2}\left\| {Z - X{Y^T}} \right\| _F^2 +{\lambda _d}{\text {Tr}}({X^T}\widetilde{\mathcal {L}}_d X) \nonumber \\{} & {} + {\lambda _t}{\text {Tr}}({Y^T}\widetilde{\mathcal {L}}_t Y) + {\lambda _l}(\left\| X \right\| _{2,1} + \left\| Y \right\| _{2,1}). \end{aligned}$$

The partial derivative of function *G* for *X* is

23 $$\begin{aligned} \frac{{\partial G}}{{\partial X}} = (Z - X{Y^T}){Y^T} + {\lambda _d}{\widetilde{\mathcal {L}}_d}X + {\lambda _l} \frac{{\partial {{\left\| X \right\| }_{2,1}}}}{{\partial X}}. \end{aligned}$$

The partial derivative of function *G* for *Y* is

24 $$\begin{aligned} \frac{{\partial G}}{{\partial Y}} = {X^T}(Z - X{Y^T}) + {\lambda _t}{\widetilde{\mathcal {L}}_t}Y + {\lambda _l} \frac{{\partial {{\left\| Y \right\| }_{2,1}}}}{{\partial Y}}, \end{aligned}$$

where $$\frac{{\partial {{\left\| X \right\| }_{2,1}}}}{{\partial X}}$$

$$= \left[ {\begin{array}{*{20}{c}} {\frac{1}{{\left\| {{X^1}} \right\| _2}}} &{} {} &{} {} &{} {} \\ {} &{} {\frac{1}{{\left\| {{X^2}} \right\| _2}}} &{} {} &{} {}&{} {} \\ {} &{} {} &{} \ddots &{} {} &{} {} \\ {} &{} {}&{} {} &{} {\frac{1}{{\left\| {{X^i}} \right\| _2}}} &{} {} \\ {} &{} {} &{} {}&{} {}&{} \ddots &{} {} &{} {} \\ {} &{} {} &{} {}&{} {}&{} {} &{} {\frac{1}{{\left\| {{X^n}} \right\| _2}}} \\ \end{array}} \right] X,$$

$$\frac{{\partial {{\left\| Y \right\| }_{2,1}}}}{{\partial Y}}$$

$$= \left[ {\begin{array}{*{20}{c}} {\frac{1}{{\left\| {{Y^1}} \right\| _2}}} &{} {} &{} {} &{} {} \\ {} &{} {\frac{1}{{\left\| {{Y^2}} \right\| _2}}} &{} {} &{} {} \\ {} &{} {} &{} \ddots &{} {} &{} {} \\ {} &{} {} &{} {} &{} {\frac{1}{{\left\| {{Y^j}} \right\| _2}}} &{} {} \\ {} &{} {} &{} {}&{}{} &{} \ddots &{} {} &{} {} \\ {} &{} {} &{} {} &{} {} &{}{} &{}{\frac{1}{{\left\| {{Y^m}} \right\| _2}}} \\ \end{array}} \right] Y,$$

For sequences ${(X^i, Y^i)}_{i \in \mathbb {N}}$, ${(m_{1}^{i}, m_{2}^{i})}_{i \in \mathbb {N}}$, ${(n_{1}^{i}, n_{2}^{i})}_{i \in \mathbb {N}}$, parameters $c _1^i$, $c_2^i$, $\beta _1^{i}$ and $\beta _2^{i}$, we can get

25 $$\begin{aligned}{} & {} \left\{ \begin{array}{c} m_{1}^{i}=X^{i}+\alpha _{1}^{i}(X^{i}-X^{i-1}), \\ n_{1}^{i}=X^{i}+\beta _{1}^{i}(X^{i}-X^{i-1}), \\ {X^{i + 1}} \in prox_{c_{1}^{i}}^{\delta _X}({m_1^i}- {\frac{1}{c_{1}^{i}} \nabla _{X}} G(n_{1}^i,Y^i) ).\\ \end{array} \right. \end{aligned}$$

26 $$\begin{aligned}{} & {} \left\{ \begin{array}{c} m_{2}^{i}=Y^{i}+\alpha _{2}^{i}(Y^{i}-Y^{i-1}), \\ n_{2}^{i}=Y^{i}+\beta _{2}^{i}(Y^{i}-Y^{i-1}), \\ {Y^{i + 1}} \in prox_{c_{2}^{i}}^{\delta _Y}({m_2^i}- {\frac{1}{c_{2}^{i}} \nabla _{Y}} G(X^{i+1},n_{2}^i) ).\\ \end{array} \right. \end{aligned}$$

The pseudocode of the algorithm (iPALM-DLMF) is shown in Algorithm 1.
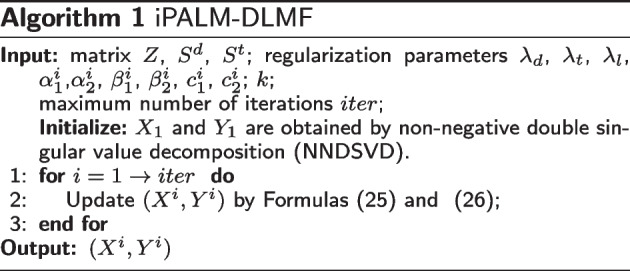


## Experiments

To evaluate the performance of DTIs prediction algorithms, 5 repetitions of 10-fold cross-validation are performed for all prediction methods. The averages 5 repetitions of 10-fold cross-validation results are used as the final test results.

The cross-validation experiments are conducted under the following two scenarios [55].

1. $CV_d$: The drugs are divided in ten folds, each fold is selected in turn as the test dataset and the other remained 9 folds are used as the training dataset. If the *i*-th drug is in the test dataset, the elements in the *i*-th row of *Z* are all set 0, which means the known interactions with tested drugs are removed from the input DTI matrix. It aims to evaluate the targeted protein prediction performance for the drugs without any known interactive targets.
2. $CV_t$: The targets are divided in ten folds, each fold is selected in turn as the test dataset and the other remained 9 folds are used as the training dataset. If the *j*-th target in the test dataset, the elements in the *j*-th column of *Z* are all set 0, which means the known interactions with tested targets are removed from the input DTI matrix. It aims to evaluate the targeting drug prediction performance for the targets without any known interactive drugs.

We use the area under receiver operating characteristic curve (AUC) and area under the precision-recall curve (AUPR) to evaluate performance of methods.

### Comparison with state-of-the-art methods

iPALM-DLMF are compared with the following eight methods, namely BLM-NII [15], WKNKN [16], RLS-WNN [14], GRMF [31], WGRMF, CMF [29], SRCMF [33] and MK-TCMF [34], where WGRMF is a weighted form of GRMF. Among them, BlM-NII, WKNKN and RLS-WNN use the neighborhood information of graph to predict DTIs, while the others are model based on matrix factorization.

#### Parameter settings

According to the original literature [31, 33, 34] and the source code of GRMF [31], we set parameters to obtain results of relevant methods. For iPALM-DLMF, according to previous research [31], grid search [56] are used to choose parameters based on the AUPR value. the regularization parameter $\lambda _l$ is selected from $\{2^{-2}, 2^{-1}, 2^{0}, 2^{1}\}$. $\lambda _d$ and $\lambda _t$ are selected from $\{0, 10^{-4}, 10^{-3}, 10^{-2}, 10^{-1}\}$. The numbers of maximum iterations are 2. *k* is 26 on NR. *k* is 49 on GPCR. rank *k* is selected from $\left\{ {50, 100} \right\}$ on IC and E. For inertial parameters $\alpha _{1}^i=\alpha _{2}^i=0.2, \beta _{1}^i=\beta _{2}^i=0.4$. $c_{1}^{i}={\left\| {{Y^i}{{({Y^i})}^T}} \right\| _F}$, $c_{2}^{i}={\left\| {{X^i}{{({X^i})}^T}} \right\| _F}$.

**Fig. 2:**
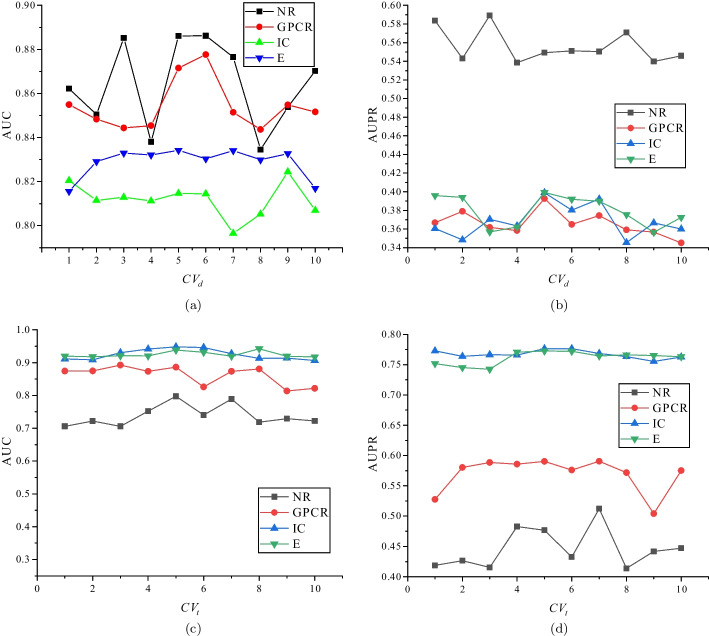
Performance of iPALM-DLMF on four benchmark datasets with different values of *K*

In order to explore the effect of performance of iPALM-DLMF with different values of *K*, we change the values of *K* and show the corresponding AUC and AUPR of iPALM-DLMF under the $CV_d$ and $CV_t$ scenario in Fig. 2. We can find from these four figures that with the increase of the values of *K*, the performance of iPALM-DLMF can not maintain stability on different datasets. As shown in Fig. 2, iPALM-DLMF is very sensitive to the value of *K*. Therefore, based on [31], we set $K=5$.

#### Prediction results

**Fig. 3:**
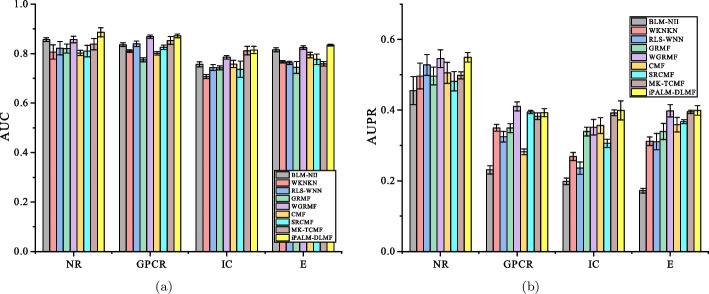
AUC values and AUPR values of the methods on the four datasets under $CV_d$.** a** Histogram with error bars of AUC.** b** Histogram with error bars of AUPR

**Fig. 4:**
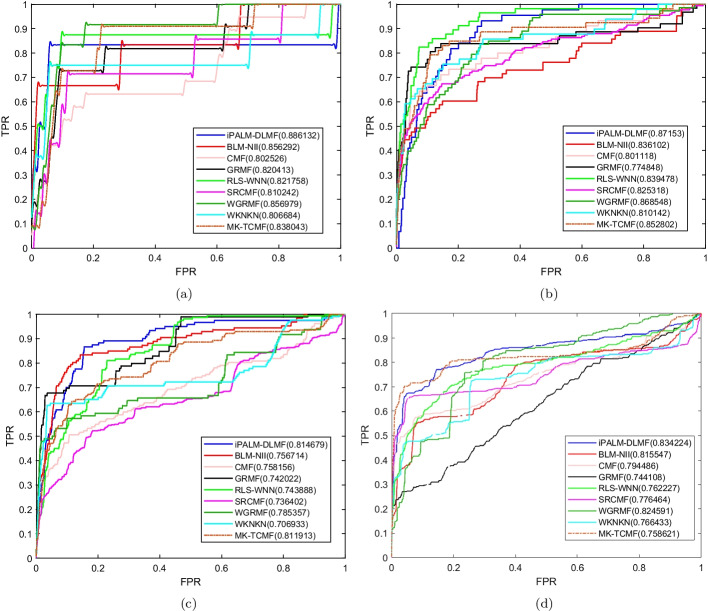
ROC curves for different methods are plotted together under $CV_d$, where subfigures **a**, **b**, **c**, **d** correspond to ROC curves on NR dataset, GPCR dataset, IC dataset, E dataset, respectively.

**Fig. 5:**
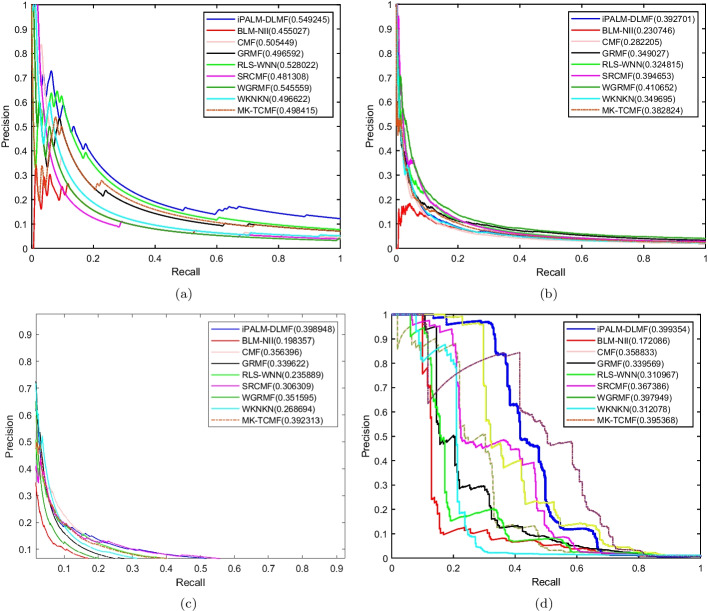
PR curves for different methods are plotted together under $CV_d$, where subfigures **a**, **b**, **c**, **d** correspond to PR curves on NR dataset, GPCR dataset, IC dataset, E dataset, respectively

Under the $CV_d$ scenario, iPALM-DLMF performs better than other methods in terms of AUC and AUPR on NR, GPCR, IC, and E datasets. The AUC values of iPALM-DLMF are 0.886132, 0.87153, 0.814679 and 0.834224 on NR, GPCR, IC, and E datasets, respectively. The AUPR values of iPALM-DLMF are 0.549245, 0.398948, and 0.399354 on NR, IC, and E datasets, respectively. On the GPCR dataset, WGRMF achieve the highest AUPR values, which are 0.410652. The AUPR value of iPALM-DLMF is 0.392701. The AUC and AUPR values of the different algorithms on the four datasets are shown in Tables 2 and 3, respectively. The AUC and AUPR histograms with error bars of different algorithms are shown in Fig. 3a and b, respectively. The receiver operating characteristic (ROC) curves and the precision-recall (PR) curves of different methods on the four datasets are shown in Figs. 4 and 5, respectively.

**Table 2:** AUC values of different algorithms under $CV_d$ scenario

Method	NR	GPCR	IC	E
BLM-NII [15]	0.856292 (0.0077)	0.836102 (0.0073)	0.756714 (0.0102)	0.815547 (0.0080)
WKNKN [16]	0.806684 (0.0289)	0.810142 (0.0048)	0.706933 (0.0079)	0.766433 (0.0050)
RLS-WNN [14]	0.821758 (0.0273)	0.839478 (0.0116)	0.743888 (0.0113)	0.762227 (0.0066)
GRMF [31]	0.820413 (0.0185)	0.774848 (0.0082)	0.742022 (0.0080)	0.744108 (0.0240)
WGRMF [31]	0.856979 (0.0135)	0.868548 (0.0065)	0.785357 (0.0070)	0.824591 (0.0071)
CMF [29]	0.802526 (0.0109)	0.801118 (0.0069)	0.758156 (0.0144)	0.794486 (0.0109)
SRCMF [33]	0.810242 (0.0227)	0.825318 (0.0093)	0.736402 (0.0329)	0.776464 (0.0214)
MK-TCMF [34]	0.838043 (0.0228)	0.852802 (0.0158)	0.811913 (0.0171)	0.758621 (0.0092)
iPALM-DLMF	**0.886132** (0.0184)	**0.87153** (0.0074)	**0.814679** (0.0150)	** 0.834224** (0.0035)

The maximum AUC on each dataset is shown in bold. Standard deviation is shown in parentheses

**Table 3:** AUPR values of different algorithms under $CV_d$ scenario

Method	NR	GPCR	IC	E
BLM-NII [15]	0.455027 (0.0395)	0.230746 (0.0118)	0.198357 (0.0091)	0.172086 (0.0068)
WKNKN [16]	0.496622 (0.0366)	0.349695 (0.0096)	0.268694 (0.0113)	0.312078 (0.0121)
RLS-WNN [14]	0.528022 (0.0294)	0.324815 (0.0149)	0.235889 (0.0176)	0.310967 (0.0232)
GRMF [31]	0.496592 (0.0252)	0.349027 (0.0129)	0.339622 (0.0124)	0.339569 (0.0227)
WGRMF [31]	0.545559 (0.0252)	**0.410652** (0.0126)	0.351595 (0.0223)	0.397949 (0.0176)
CMF [29]	0.505449 (0.0299)	0.282205 (0.0081)	0.356396 (0.0227)	0.358833 (0.0205)
SRCMF [33]	0.481308 (0.0273)	0.394653 (0.0049)	0.306309 (0.0116)	0.367386 (0.0054)
MK-TCMF [34]	0.498415 (0.0097)	0.382824 (0.009)	0.392313 (0.0079)	0.395368 (0.0044)
iPALM-DLMF	**0.549245** (0.0137)	0.392701 (0.0111)	**0.398948** (0.0269)	**0.399354** (0.0136)

The maximum AUPR on each dataset is shown in bold. Standard deviation is shown in parentheses

**Fig. 6:**
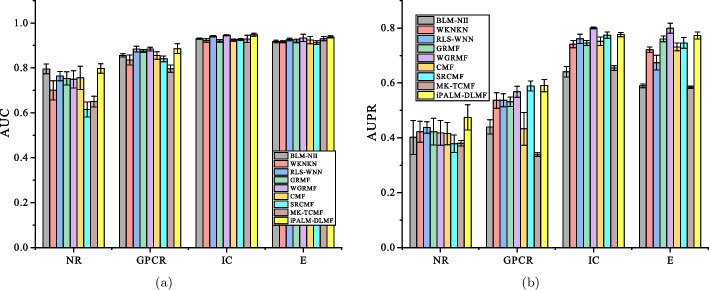
AUC values and AUPR values of the methods on the four datasets under $CV_t$. **a** Histogram with error bars of AUC. **b** Histogram with error bars of AUPR

**Fig. 7:**
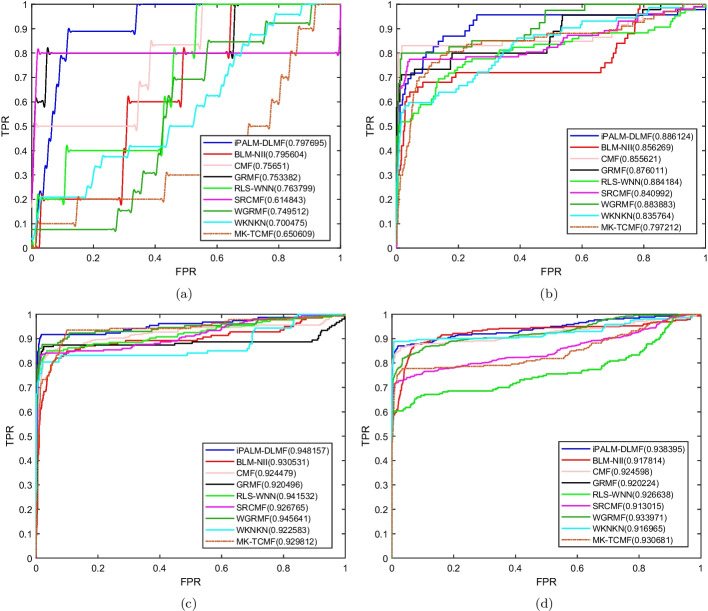
ROC curves for different methods are plotted together under $CV_t$, where subfigures **a**, **b**, **c**, **d** correspond to ROC curves on NR dataset, GPCR dataset, IC dataset, E dataset, respectively

**Fig. 8:**
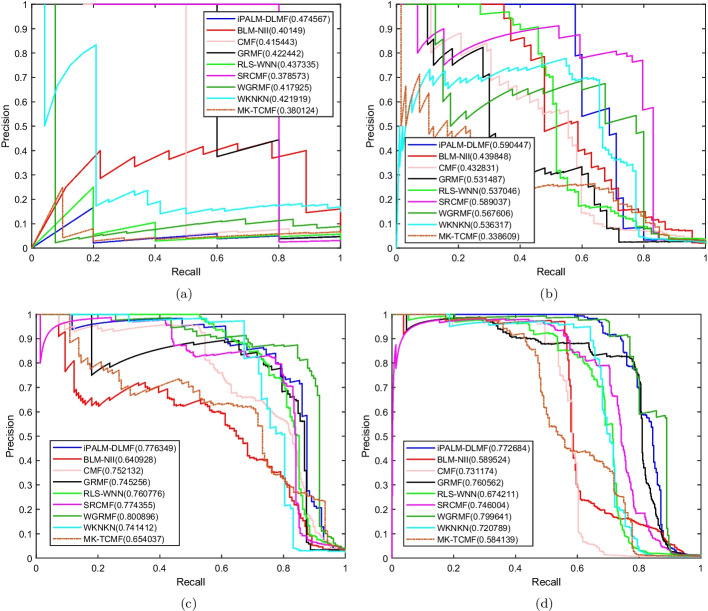
PR curves for different methods are plotted together under $CV_t$, where subfigures **a**, **b**, **c**, **d** correspond to PR curves on NR dataset, GPCR dataset, IC dataset, E dataset, respectively

Under the $CV_t$ scenario, the AUC of iPALM-DLMF are higher than the other methods on the four datasets. The AUC values of iPALM-DLMF are 0.797695, 0.886124, 0.948157 and 0.938395 on NR, GPCR, IC, and E datasets, respectively. The AUPR values of iPALM-DLMF on NR and GPCR datasets are 0.474567 and 0.590447, respectively. On the IC and E dataset, WGRMF achieve the highest AUPR values, which are 0.800896 and 0.799641, respectively. The AUPR value of iPALM-DLMF is 0.776349 and 0.772684 on the IC and E dataset, respectively. The AUC values and AUPR values of different algorithms on the four datasets are shown in Table 4 and Table 5, respectively. The AUC and AUPR histograms with error bars of different algorithms are shown in Fig. 6a and b, respectively. ROC and PR curves of different algorithms are shown in Fig. 7 and Fig. 8 on the four datasets, respectively.

**Table 4:** AUC values of different algorithms under $CV_t$ scenario

Method	NR	GPCR	IC	E
BLM-NII [15]	0.795604 (0.0217)	0.856269 (0.0071)	0.930531 (0.0029)	0.917814 (0.0056)
WKNKN [16]	0.700475 (0.0430)	0.835764 (0.0217)	0.922583 (0.0079)	0.916965 (0.0042)
RLS-WNN [14]	0.763799 (0.0208)	0.884184 (0.0128)	0.941532 (0.0031)	0.926638 (0.0053)
GRMF [31]	0.753382 (0.0293)	0.876011 (0.0063)	0.920496 (0.0060)	0.920224 (0.0074)
WGRMF [31]	0.749512 (0.0384)	0.883883 (0.0083)	0.945641 (0.0024)	0.933971 (0.0161)
CMF [29]	0.75651 (0.0520)	0.855621 (0.0164)	0.924479 (0.0051)	0.924598 (0.0161)
SRCMF [33]	0.614843 (0.0333)	0.840992 (0.0127)	0.926765 (0.0049)	0.913015 (0.0082)
MK-TCMF [34]	0.650609 (0.0238)	0.797212 (0.0164)	0.929812 (0.0165)	0.930681 (0.0092)
iPALM-DLMF	**0.797695** (0.0214)	**0.886124** (0.0218)	**0.948157** (0.0069)	**0.938395** (0.0048)

The maximum AUC on each dataset is shown in bold. Standard deviation is shown in parentheses

**Table 5:** AUPR values of different algorithms under $CV_t$ scenario

Method	NR	GPCR	IC	E
BLM-NII [15]	0.40149 (0.0618)	0.439848 (0.0259)	0.640928 (0.0191)	0.589524 (0.0069)
WKNKN [16]	0.421919 (0.0382)	0.536317 (0.0281)	0.741412 (0.0131)	0.720789 (0.0100)
RLS-WNN [14]	0.437335 (0.0206)	0.537046 (0.0235)	0.760776 (0.0169)	0.674211 (0.0266)
GRMF [31]	0.422442 (0.0486)	0.531487 (0.0175)	0.745256 (0.0091)	0.760562 (0.0100)
WGRMF [31]	0.417925 (0.0447)	0.567606 (0.0201)	**0.800896** (0.0036)	**0.799641** (0.0185)
CMF [29]	0.415443 (0.0407)	0.432831 (0.0596)	0.752132 (0.0154)	0.731174 (0.0140)
SRCMF [33]	0.378573 (0.0318)	0.589037 (0.0183)	0.774355 (0.0117)	0.746004 (0.0198)
MK-TCMF [34]	0.380124 (0.0098)	0.338609 (0.0071)	0.654037 (0.0086)	0.584139 (0.005)
iPALM-DLMF	** 0.474567** (0.0461)	**0.590447** (0.0225)	0.776349 (0.0076)	0.772684 (0.0126)

The maximum AUPR on each dataset is shown in bold. Standard deviation is shown in parentheses

### Ablation experiments

In order to determine the effect of several techniques on performance in our proposed iPALM-DLMF, we separately assess the performance of iPALM-DLMF, iPALM-DLMF (without NNDSVD, i.e. using SVD in the initialization stage of matrix factorization), iPALM-DLMF ( $\lambda _d$=0, i.e. the graph regularization term for drugs is not used), iPALM-DLMF ( $\lambda _t$=0, i.e. the graph regularization term for targets is not used), iPALM-DLMF ( $\lambda _l$=0, i.e. $L_{2,1}$ norm graph regularization is not used) and PALM-GRMF (i.e. inertial forces is not used). The results of above settings are shown in Tables  6, 7, 8, and 9.

In Tables 6, 7, 8, and 9, iPALM-DLMF have better performance than other settings. In $CV_d$, when NNDSVD are used in the initialization stage of matrix factorization, the AUC values have increased by 0.6%, 1.8%, 2% on NR, GPCR and E datasets, respectively, and the AUC values have decreased by 1.3% on IC datasets. The AUPR values have increased by 2.2%, 11.5%, 6%, 4% on NR, GPCR, IC and E datasets, respectively. In $CV_t$, using NNDSVD, The AUC values have increased by 6%, 6%, 3%, 1.6% on NR, GPCR, IC and E datasets, respectively. The AUPR values have increased by 6.5%, 9.7%, 1.5% and 3.4% on NR, GPCR, IC and E data sets, respectively. Experimental results show that using NNDSVD in the initial stage of matrix factorization can improve the ability of the algorithm to predict DTIs.

When we use regularization terms for drugs and targets, iPALM-DLMF has the good prediction performance in $CV_d$ and $CV_t$. In $CV_d$, when $\lambda _d=0$, the values of AUC and AUPR of iPALM-DLMF are significantly decreased. The AUC values have decreased by 30%, 27%, 27%, 34% on NR, GPCR, IC and E datasets, respectively. The AUPR values have decreased by 75%, 82%, 87%, 96% on NR, GPCR, IC and E datasets, respectively. Similarly, in $CV_t$, if the graph regularization terms for targets is not used, the performances of iPALM-DLMF is significantly decreased too. When $\lambda _t=0$, the AUC values have decreased by 37%, 38%, 39%, 42% on NR, GPCR, IC and E datasets, respectively. The AUPR values have decreased by 79%, 92%, 91%, 98% on NR, GPCR, IC and E datasets, respectively. When $\lambda _t=0$, these results show that regularization terms for drugs and targets contribute the improvement of DTIs prediction performance of iPALM-DLMF significantly.

In $CV_d$, when $\lambda _l=0$, the values of AUC and AUPR of iPALM-DLMF are decreased. The AUC values have decreased by 3%, 2%, 0.4%, 1.1% on NR, GPCR, IC and E datasets, respectively. The AUPR values have decreased by 1.9%, 7.2%, 4.8%, 7% on NR, GPCR, IC and E datasets, respectively. Similarly, in $CV_t$, when $\lambda _l=0$, the AUC values have decreased by 8.6%, 6.4%, 3.5%, 1.8% on NR, GPCR, IC and E datasets, respectively. The AUPR values have decreased by 0.5%, 11%, 1.1%, 2.8% on NR, GPCR, IC and E datasets, respectively. When $\lambda _l=0$, these results show that $L_{2,1}$ regularization terms for drugs and targets contribute the improvement of DTIs prediction performance of iPALM-DLMF.

When inertial terms is not used in iPALM-DLMF, the values of AUC and AUPR of iPALM-DLMF are decreased under $CV_d$ scenario. The AUC values have decreased by 3.8%, 0.7%, 1.1%, 1.8% on NR, GPCR, IC and E datasets, respectively. The AUPR values have decreased by 0.3%, 3.8%, 8.4%, 5.2% on NR, GPCR, IC and E datasets, respectively. Similarly, the AUC values have decreased by 7.8%, 6.5%, 4.8%, 1.1% on NR, GPCR, IC and E datasets in $CV_t$, respectively. The AUPR values have decreased by 9.3%, 12.8%, 1.3%, 0.3% on NR, GPCR, IC and E datasets, respectively. These results show that inertial terms contribute the improvement of DTIs prediction performance of iPALM-DLMF.

**Table 6:** AUC values of different algorithms under $CV_d$ scenario

Method	NR	GPCR	IC	E
iPALM-DLMF	**0.886132** (0.0184)	**0.87153** (0.0074)	0.814679 (0.0150)	** 0.834224** (0.0035)
iPALM-DLMF (without NNDSVD)	0.880843 (0.0264)	0.855862 (0.0074)	**0.825428** (0.0167)	0.817143 (0.0085)
iPALM-DLMF ( $\lambda _d$=0)	0.620998 (0.0543)	0.639598 (0.0268)	0.592975 (0.0140)	0.547534 (0.0098)
iPALM-DLMF ( $\lambda _t$=0)	0.831302 (0.0180)	0.844522 (0.0048)	0.815958 (0.0140)	0.805771 (0.0049)
iPALM-DLMF ( $\lambda _l$=0)	0.859537 (0.0099)	0.853745 (0.0094)	0.811177 (0.0104)	0.824733 (0.0111)
PALM-DLMF	0.852378 (0.0262)	0.865187 (0.0072)	0.805869 (0.0062)	0.81903 (0.0031)

The maximum AUC on each dataset is shown in bold. Standard deviation is shown in parentheses

**Table 7:** AUPR values of different algorithms under $CV_d$ scenario

Method	NR	GPCR	IC	E
iPALM-DLMF	**0.549245** (0.0137)	**0.392701** (0.0111)	**0.398948** (0.0269)	**0.399354** (0.0136)
iPALM-DLMF (without NNDSVD)	0.537198 (0.0237)	0.34756 (0.0049)	0.374837 (0.0212)	0.383282 (0.0172)
iPALM-DLMF ( $\lambda _d$=0)	0.135244 (0.0152)	0.072055 (0.0102)	0.053728 (0.0031)	0.016293 (0.0011)
iPALM-DLMF ( $\lambda _t$=0)	0.491092 (0.0268)	0.347821 (0.0059)	0.363915 (0.0201)	0.35153 (0.0038)
iPALM-DLMF ( $\lambda _l$=0)	0.539008 (0.0226)	0.364441 (0.0100)	0.37975 (0.0158)	0.371432 (0.0277)
PALM-DLMF	0.547462 (0.0326)	0.377908 (0.0103)	0.3656 (0.0079)	0.378476 (0.0083)

The maximum AUPR on each dataset is shown in bold. Standard deviation is shown in parentheses

**Table 8:** AUC values of different algorithms under $CV_t$ scenario

Method	NR	GPCR	IC	E
iPALM-DLMF	**0.797695** (0.0214)	**0.886124** (0.0218)	**0.948157** (0.0069)	**0.938395** (0.0048)
iPALM-DLMF (without NNDSVD)	0.749641 (0.0249)	0.832083 (0.0200)	0.919673 (0.0066)	0.923762 (0.0084)
iPALM-DLMF ( $\lambda _d$ =0)	0.559851 (0.0288)	0.801559 (0.0261)	0.90617 (0.0084)	0.903253 (0.0084)
iPALM-DLMF ( $\lambda _t$=0)	0.498876 (0.0290)	0.553367 (0.0232)	0.582471 (0.0166)	0.547481 (0.0136)
iPALM-DLMF ( $\lambda _l$=0)	0.72897 (0.0133)	0.828976 (0.0184)	0.914893 (0.0066)	0.921384 (0.0054)
PALM-DLMF	0.735579 (0.0248)	0.828103 (0.0175)	0.902373 (0.0009)	0.928485 (0.0069)

The maximum AUC on each dataset is shown in bold. Standard deviation is shown in parentheses

**Table 9:** AUPR values of different algorithms under $CV_t$ scenario

Method	NR	GPCR	IC	E
iPALM-DLMF	**0.47678 ** (0.0461)	**0.590447** (0.0225)	**0.776349 (0.0076)**	**0.772684** (0.0126)
iPALM-DLMF (without NNDSVD)	0.445602 (0.0205)	0.532892 (0.0346)	0.764708 (0.0091)	0.745737 (0.0173)
iPALM-DLMF ($\lambda _d$=0)	0.393418 (0.0226)	0.53539 (0.0257)	0.769829 (0.0190)	0.751815 (0.0132)
iPALM-DLMF ( $\lambda _t$=0)	0.101816 (0.0137)	0.049806 (0.0048)	0.072554 (0.0109)	0.018001 (0.0018)
iPALM-DLMF ( $\lambda _l$=0)	0.474567 (0.0287)	0.525707 (0.0335)	0.767536 (0.0137)	0.751271 (0.0068)
PALM-DLMF	0.432608 (0.0479)	0.514862 (0.0368)	0.766604 (0.0066)	0.770734 (0.0090)

The maximum AUPR on each dataset is shown in bold. Standard deviation is shown in parentheses

### Case studies

To further evaluate the ability of iPALM-DLMF to find new targets for a drug and new drugs for a target in practice, two case studies concerning the drug gabapentin and the target prostaglandin-endoperoxide synthase 2 were conducted. Furthermore, we also conducted experiments according to [23].

In the first case study, we predicted targets that interact with the drug gabapentin on the IC dataset using iPALM-DLMF. Gabapentin (GBP) is an antiepileptic drug, which is an amino acid. In the mechanism of action, gabapentin (GBP) is different from other anticonvulsant drugs which makes identifying interaction target for GBP more complicated [57]. The known interactions of gabapentin with targets were deleted from the training dataset, and the candidate targets of gabapentin predicted by iPALM-DLMF were prioritized according to the prediction scores. At last, the top 50 highest-scoring predicted targets were picked out to be validated using the original database [12]. The results showed that 46 targets had evidences to drug GBP among the predicted 50 drugs. The detailed results of the predictions are shown in Table 10.

In the second case study, we predicted candidate drugs for the target prostaglandin-endoperoxide synthase 2 (PTGS2) on the E dataset and aimed to assess the ability of iPALM-DLMF to predict candidate drugs for targets with no known targeting drugs. PTGS2 expression has been validated to be associated with colorectal cancer. However, PTGS2 and prostaglandin-endoperoxide synthase 1 are confused in colorectal cancer pathology and therapy. The known interactions of PTGS2 with drugs is essential in clinic [58]. The known interactions of PTGS2 with drugs were removed from the training dataset, and the candidate drugs of PTGS2 predicted by iPALM-DLMF were prioritized according to the prediction scores. The top 50 highest-scoring predicted drugs were selected to be validated against original database [12] and literatures. Among the predicted 50 drugs, 47 drugs had evidences to target PTGS2, where pentoxifylline, mesalamine, suprofen, mofezolac and sulfinpyrazone have been validated to interact with PTGS2 by literature [59–63], respectively. This means that iPALM-DLMF have good performance for new predicted interactions. The detailed results of the case study are shown in Table 11.

**Table 10:** Top 50 predicted targets of Gabapentin by iPALM-DLMF on the IC dataset

Rank	Name of targets	ID	Evidence
1	Calcium voltage-gated channel subunit alpha1 H	hsa8912	Confirmed
2	Calcium voltage-gated channel auxiliary subunit gamma 1	hsa786	Confirmed
3	Calcium voltage-gated channel auxiliary subunit alpha2delta 4	hsa93589	Confirmed
4	Calcium voltage-gated channel auxiliary subunit beta 2	hsa783	Confirmed
5	Calcium voltage-gated channel subunit alpha1 I	hsa8911	Confirmed
6	Calcium voltage-gated channel subunit alpha1 G	hsa8913	Confirmed
7	Calcium voltage-gated channel subunit alpha1 C	hsa775	Confirmed
8	Calcium voltage-gated channel subunit alpha1 F	hsa778	Confirmed
9	Calcium voltage-gated channel subunit alpha1 D	hsa776	Confirmed
10	Calcium voltage-gated channel auxiliary subunit alpha2delta 1	hsa781	Confirmed
11	Calcium voltage-gated channel subunit alpha1 S	hsa779	Confirmed
12	Calcium voltage-gated channel subunit alpha1 E	hsa777	Confirmed
13	Calcium voltage-gated channel subunit alpha1 A	hsa773	Confirmed
14	Calcium voltage-gated channel auxiliary subunit beta 1	hsa782	Confirmed
15	Calcium voltage-gated channel subunit alpha1 B	hsa774	Confirmed
16	Calcium voltage-gated channel auxiliary subunit beta 4	hsa785	Confirmed
17	Calcium voltage-gated channel auxiliary subunit alpha2delta 3	hsa55799	Confirmed
18	Calcium voltage-gated channel auxiliary subunit gamma 2	hsa10369	Confirmed
19	Calcium voltage-gated channel auxiliary subunit alpha2delta 2	hsa9254	Confirmed
20	Calcium voltage-gated channel auxiliary subunit gamma 4	hsa27092	Confirmed
21	Calcium voltage-gated channel auxiliary subunit beta 3	hsa784	Confirmed
22	Inositol 1,4,5-trisphosphate receptor type 1	hsa3708	Confirmed
23	Inositol 1,4,5-trisphosphate receptor type 3	hsa3710	Confirmed
24	Transient receptor potential cation channel subfamily A member 1	hsa8989	Confirmed
25	Calcium voltage-gated channel auxiliary subunit gamma 7	hsa59284	Confirmed
26	Transient receptor potential cation channel subfamily V member 6	hsa55503	Confirmed
27	Polycystin 1	hsa5310	Confirmed
28	Sodium channel epithelial 1 subunit alpha	hsa6337	Confirmed
29	Sodium channel epithelial 1 subunit gamma	hsa6340	Confirmed
30	Sodium channel epithelial 1 subunit delta	hsa6339	Confirmed
31	Sodium channel epithelial 1 subunit beta	hsa6338	Confirmed
32	Acid sensing ion channel subunit family member 5	hsa51802	Confirmed
33	Acid sensing ion channel subunit family member 4	hsa55515	Confirmed
34	Acid sensing ion channel subunit 3	hsa9311	Confirmed
35	Sodium voltage-gated channel beta subunit 3	hsa55800	Confirmed
36	Sodium voltage-gated channel beta subunit 1	hsa6324	Confirmed
37	Sodium voltage-gated channel beta subunit 4	hsa6330	Confirmed
38	Sodium voltage-gated channel alpha subunit 7	hsa6332	Confirmed
39	Ryanodine receptor 3	hsa6263	Confirmed
40	Ryanodine receptor 2	hsa6262	Confirmed
41	Ryanodine receptor 1	hsa6261	Confirmed
42	Sodium voltage-gated channel alpha subunit 10	hsa6336	Confirmed
43	Sodium voltage-gated channel alpha subunit 3	hsa6328	Confirmed
44	Sodium voltage-gated channel alpha subunit 4	hsa6329	Confirmed
45	Sodium voltage-gated channel alpha subunit 5	hsa6331	Confirmed
46	glutamate ionotropic receptor NMDA type subunit 3A	hsa116443	Confirmed
47	Potassium two pore domain channel subfamily K member 13	hsa56659	Unknown
48	Potassium two pore domain channel subfamily K member 5	hsa8645	Unknown
49	Potassium Calcium-activated channel subfamily N member 4	hsa3783	Unknown
50	ATP binding cassette subfamily C member 9	hsa10060	Unknown

**Table 11:** Top 50 predicted drugs of prostaglandin-endoperoxide synthase 2 by iPALM-DLMF on the E dataset

Rank	Name of targets	ID	Evidence	Rank	Name of targets	ID	Evidence
1	Ketoprofen	D00132	Confirmed	26	Meclofenamic acid	D02341	Confirmed
2	Indomethacin	D00141	Confirmed	27	Tenoxicam	D01767	Confirmed
3	Naproxen	D00118	Confirmed	28	Sodium salicylate	D00566	Confirmed
4	Phenylbutazone	D00510	Confirmed	29	Lornoxicam	D01866	Confirmed
5	Ibuprofen	D00126	Confirmed	30	Valdecoxib	D02709	Confirmed
6	Caffeine	D00528	Unknown	31	Diclofenac potassium	D00903	Confirmed
7	Pentoxifylline	D00501	Confirmed	32	Diclofenac sodium	D00904	Confirmed
8	Milrinone	D00417	Unknown	33	Lumiracoxib	D03714	Confirmed
9	Mesalamine	D00377	Confirmed	34	Etoricoxib	D03710	Confirmed
10	Acetaminophen	D00217	Confirmed	35	Meloxicam	D00969	Confirmed
11	Ciclopirox olamine	D01364	Unknown	36	Piroxicam	D00127	Confirmed
12	Sulindac	D00120	Confirmed	37	Tolfenamic acid	D01183	Confirmed
13	Celecoxib	D00567	Confirmed	38	Alminoprofen	D01513	Confirmed
14	Fenoprofen	D02350	Confirmed	39	Ampiroxicam	D01397	Confirmed
15	Suprofen	D00452	Confirmed	40	Diflunisal	D00130	Confirmed
16	Flurbiprofen	D00330	Confirmed	41	Parecoxib	D03716	Confirmed
17	Mofezolac	D01718	Confirmed	42	Nabumetone	D00425	Confirmed
18	Acemetacin	D01582	Confirmed	43	Indometacin farnesil	D01565	Confirmed
19	Naproxen sodium	D00970	Confirmed	44	Magnesium salicylate	D00827	Confirmed
20	Mefenamic acid	D00151	Confirmed	45	Pranoprofen	D01578	Confirmed
21	Meclofenamate sodium	D00169	Confirmed	46	Tolmetin sodium	D00158	Confirmed
22	Tolmetin	D02355	Confirmed	47	Sulfinpyrazone	D00449	Confirmed
23	Tiaprofenic acid	D01325	Confirmed	48	Flurbiprofen axetil	D01475	Confirmed
24	Flurbiprofen sodium	D02290	Confirmed	49	Choline salicylate	D00810	Confirmed
25	Indomethacin sodium	D02110	Confirmed	50	Ketorolac tromethamine	D00813	Confirmed

**Fig. 9:**
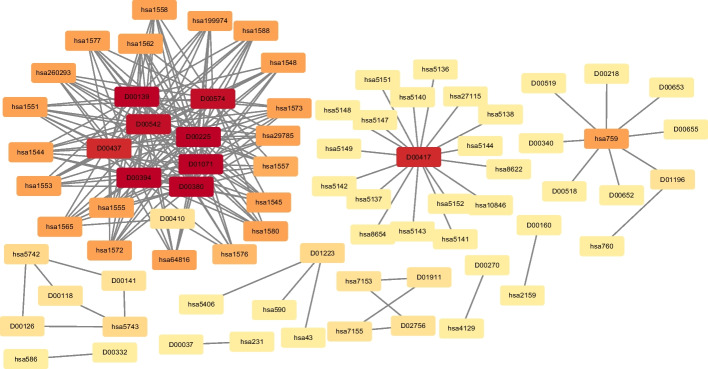
Network visualization of the drug–target interactions predicted by iPALM-DLMF

According [23], the whole heterogeneous network (in which drug and targets have at least one known interacting pair) was regarded as training data on the E dataset. We removed 80000 protein-protein interactions from the target proteins network in training data. Among the top 200 highest-scoring predictions, we found that all of them can also be supported by the original database [12]. Networks of the predicted drug–target interactions are shown in Fig. 9.

## Conclusion

It is important to ensure sparseness of the matrices obtained by non-negative matrix factorization to find the novel usage of drugs in drug research. In this paper, we propose a matrix factorization based method, iPALM-DLMF, to predict interactions between drugs and targets. iPALM-DLMF uses graph dual regularization terms to capture structural information from the drug similarity matrix and the target similarity matrix. At the same time, $L_{2,1}$ norm regularization terms is used to ensure sparseness of the matrices obtained by non-negative matrix factorization. Finally, an inertial proximal alternating linearized minimization algorithm is used to solve the matrix factorization with graph dual regularization terms and $L_{2,1}$ norm regularization terms. Extensive experiments show that iPALM-DLMF outperforms the state-of-the-art methods in predicting DTIs.

As a kind of gradient descent methods, iPALM-DLMF can converge to KKT point. In the future, we are interested in using the idea of multi-objective particle swarm optimization [64] and fixed-point iterative method [65] to obtain a accurate solution in DTIs prediction models. At that time, more attention should be paid to synergistic drug combinations prediction problem [66].

### Supplementary Information

**Additional file 1.** The supplementary material for iPALM-DLMF.

**Additional file 2.** iPALM-DLMF + appendix.

## Data Availability

iPALM-DLMF is implemented in Matlab and freely available to the public on https://github.com/zhang340jj/iPALM-DLMF. The contents of the appendix include some description of symbols, the detailed steps of NNDSVD and the derivation of formula 17.

## References

[1] Paul SM, Mytelka DS, Dunwiddie CT, Persinger CC, Munos BH, Lindborg SR, Schacht AL (2010). How to improve R &D productivity: the pharmaceutical industry’s grand challenge. Nat Rev Drug Discov.

[2] Maryam B, Elyas S, Kai W, Sartor MA, Zaneta NC, Kayvan N (2020). Machine learning approaches and databases for prediction of drug–target interaction: a survey paper. Brief Bioinform.

[3] Gorgulla C, Boeszoermenyi A, Wang Z-F, Fischer PD, Coote PW, Padmanabha Das KM, Malets YS, Radchenko DS, Moroz YS, Scott DA, Fackeldey K, Hoffmann M, Iavniuk I, Wagner G, Arthanari H (2020). An open-source drug discovery platform enables ultra-large virtual screens. Nature.

[4] Chu Z, Huang F, Fu H, Quan Y, Zhou X, Liu S, Zhang W (2022). Hierarchical graph representation learning for the prediction of drug–target binding affinity. Inf Sci.

[5] Su X, Hu P, Yi H, You Z, Hu L (2023). Predicting drug–target interactions over heterogeneous information network. IEEE J Biomed Health Inform.

[6] Nguyen T, Le H, Quinn TP, Nguyen T, Le TD, Venkatesh S (2020). GraphDTA: predicting drug-target binding affinity with graph neural networks. Bioinformatics.

[7] Abbasi K, Razzaghi P, Poso A, Amanlou M, Ghasemi JB, Masoudi-Nejad A (2020). DeepCDA: deep cross-domain compound–protein affinity prediction through LSTM and convolutional neural networks. Bioinformatics.

[8] Chen R, Liu X, Jin S, Lin J, Liu J (2018). Machine learning for drug–target interaction prediction. Molecules.

[9] Keiser MJ, Roth BL, Armbruster BN, Ernsberger P, Irwin JJ, Shoichet BK (2007). Relating protein pharmacology by ligand chemistry. Nat Biotechnol.

[10] Sachdev K, Sachd MK (2019). A comprehensive review of feature based methods for drug target interaction prediction. J Biomed Inform.

[11] Cheng AC, Coleman RG, Smyth KT, Cao Q, Soulard P, Caffrey DR, Salzberg AC, Huang ES (2007). Structure-based maximal affinity model predicts small-molecule druggability. Nat Biotechnol.

[12] Yamanishi Y, Araki M, Gutteridge A, Honda W, Kanehisa M (2008). Prediction of drug–target interaction networks from the integration of chemical and genomic spaces. Bioinformatics.

[13] Bleakley K, Yamanishi Y (2009). Supervised prediction of drug–target interactions using bipartite local models. Bioinformatics.

[14] Twan VL, Nabuurs SB, Elena M (2011). Gaussian interaction profile kernels for predicting drug–target interaction. Bioinformatics.

[15] Mei JP, Kwoh CK, Yang P, Li XL, Zheng J (2013). Drug–target interaction prediction by learning from local information and neighbors. Bioinformatics.

[16] Twan VL, Elena M, Peter C (2013). Predicting drug–target interactions for new drug compounds using a weighted nearest neighbor profile. PLoS ONE.

[17] Ding Y, Tang J, Guo F (2020). Identification of drug–target interactions via fuzzy bipartite local model. Neural Comput Appl.

[18] Wang H, Huang F, Xiong Z, Zhang W (2022). A heterogeneous network-based method with attentive meta-path extraction for predicting drug–target interactions. Brief Bioinform.

[19] Dehghan A, Razzaghi P, Abbasi K, Gharaghani S (2023). TripletMultiDTI: multimodal representation learning in drug–target interaction prediction with triplet loss function. Expert Syst Appl.

[20] Ye Q, Hsieh C-Y, Yang Z, Kang Y, Chen J, Cao D, He S, Hou T (2021). A unified drug–target interaction prediction framework based on knowledge graph and recommendation system. Nat Commun.

[21] Zhao B-W, Wang L, Hu P-W, Wong L, Su X, Wang B-Q, You Z-H, Hu L (2023). Fusing higher and lower-order biological information for drug repositioning via graph representation learning. IEEE Trans Emerg Top Comput.

[22] Lan W, Wang J, Li M, Liu J, Li Y, Wu F-X, Pan Y (2016). Predicting drug–target interaction using positive-unlabeled learning. Neurocomputing.

[23] Luo Y, Zhao X, Zhou J, Yang J, Zhang Y, Kuang W, Peng J, Chen L, Zeng J (2017). A network integration approach for drug–target interaction prediction and computational drug repositioning from heterogeneous information. Nat Commun.

[24] Liu Z, Chen Q, Lan W, Pan H, Hao X, Pan S (2021). GADTI: graph autoencoder approach for DTI prediction from heterogeneous network. Front Genet.

[25] Rifaioglu AS, Atalay V, Martin M, Cetin-Atalay R, Doğan T (2020). DEEPScreen: high performance drug–target interaction prediction with convolutional neural networks using 2-D structural compound representations. Chem Sci.

[26] Yazdani-Jahromi M, Yousefi N, Tayebi A, Kolanthai E, Neal CJ, Seal S, Garibay OO (2022). AttentionSiteDTI: an interpretable graph-based model for drug–target interaction prediction using NLP sentence-level relation classification. Brief Bioinform.

[27] Gönen M (2012). Predicting drug-target interactions from chemical and genomic kernels using Bayesian matrix factorization. Bioinformatics.

[28] Bolgár B, Antal P (2017). VB-MK-lMF: fusion of drugs, targets and interactions using variational Bayesian multiple kernel logistic matrix factorization. BMC Bioinform.

[29] Zheng X, Ding H, Mamitsuka H, Zhu S. Collaborative matrix factorization with multiple similarities for predicting drug-target interactions. In: Proceedings of the 19th ACM SIGKDD international conference on knowledge discovery and data mining, pp. 1025–1033 (2013).

[30] Liu Y, Wu M, Miao C, Zhao P, Li X-L (2016). Neighborhood regularized logistic matrix factorization for drug–target interaction prediction. PLoS Comput Biol.

[31] Ezzat A, Zhao P, Wu M, Li X, Kwoh CK (2017). Drug–target interaction prediction with graph regularized matrix factorization. IEEE/ACM Trans Comput Biol Bioinform (TCBB).

[32] Cui Z, Gao YL, Liu JX, Dai LY, Yuan SS (2019). L2,1-GRMF: an improved graph regularized matrix factorization method to predict drug–target interactions. BMC Bioinform.

[33] Gao L-G, Yang M-Y, Wang J-X (2021). Collaborative matrix factorization with soft regularization for drug–target interaction prediction. J Comput Sci Technol.

[34] Ding Y, Tang J, Guo F, Zou Q (2022). Identification of drug–target interactions via multiple kernel-based triple collaborative matrix factorization. Brief Bioinform.

[35] Takane Y, Young FW, de Leeuw J (1977). Nonmetric individual differences multidimensional scaling: an alternating least squares method with optimal scaling features. Psychometrika.

[36] Seung D, Lee L (2001). Algorithms for non-negative matrix factorization. Adv Neural Inf Process Syst.

[37] Zhang Y. An alternating direction algorithm for nonnegative matrix factorization. Technical report. 2010

[38] Pock T, Sabach S (2016). Inertial proximal alternating linearized minimization (iPALM) for nonconvex and nonsmooth problems. SIAM J Imag Sci.

[39] Boutsidis C, Gallopoulos E (2008). SVD based initialization: a head start for nonnegative matrix factorization. Pattern Recogn.

[40] Schomburg I, Chang A, Ebeling C, Gremse M, Heldt C, Huhn G, Schomburg D (2004). Brenda, the enzyme database: updates and major new developments. Nucleic Acids Res.

[41] Kanehisa M, Goto S, Hattori M, Aoki-Kinoshita KF, Itoh M, Kawashima S, Katayama T, Araki M, Hirakawa M (2006). From genomics to chemical genomics: new developments in KEGG. Nucleic Acids Res.

[42] Günther S, Kuhn M, Dunkel M, Campillos M, Senger C, Petsalaki E, Ahmed J, Urdiales EG, Gewiess A, Jensen LJ, Schneider R, Skoblo R, Russell RB, Bourne PE, Bork P, Preissner R (2007). Supertarget and matador: resources for exploring drug–target relationships. Nucleic Acids Res.

[43] Wishart DS, Knox C, Guo AC, Cheng D, Shrivastava S, Tzur D, Gautam B, Hassanali M (2007). DrugBank: a knowledgebase for drugs, drug actions and drug targets. Nucleic Acids Res.

[44] Hattori M, Okuno Y, Goto S, Kanehisa M (2003). Development of a chemical structure comparison method for integrated analysis of chemical and genomic information in the metabolic pathways. J Am Chem Soc.

[45] Smith T, Waterman M (1981). Identification of common molecular subsequences. J Mol Biol.

[46] Wang Y, Zhang Y (2013). Nonnegative matrix factorization: a comprehensive review. IEEE Trans Knowl Data Eng.

[47] Cai D, He X, Han J, Huang TS (2011). Graph regularized non-negative matrix factorization for data representation. IEEE Trans Pattern Anal Mach Intell.

[48] Shang FH, Jiao LC, Wang F (2012). Graph dual regularization non-negative matrix factorization for co-clustering. Pattern Recogn.

[49] Bolte J, Sabach S, Teboulle M (2014). Proximal alternating linearized minimization for nonconvex and nonsmooth problems. Math Program.

[50] Lions P-L, Mercier B (1979). Splitting algorithms for the sum of two nonlinear operators. SIAM J Numer Anal.

[51] Combettes PL, Wajs VR (2005). Signal recovery by proximal forward-backward splitting. Multiscale Model Simul.

[52] Alvarez F, Attouch H (2001). An inertial proximal method for maximal monotone operators via discretization of a nonlinear oscillator with damping. Set Valued Anal.

[53] Polyak BT (1964). Some methods of speeding up the convergence of iteration methods. USSR Comput Math Math Phys.

[54] Ochs P, Chen Y, Brox T, Pock T (2014). iPiano: inertial proximal algorithm for nonconvex optimization. SIAM J Imag Sci.

[55] Pahikkala T, Airola A, Pietila S, Shakyawar S, Szwajda A, Tang J, Aittokallio T (2015). Toward more realistic drug–target interaction predictions. Brief Bioinform.

[56] Bergstra J, Bengio Y (2012). Random search for hyper-parameter optimization. J Mach Learn Res.

[57] Taylor CP, Gee NS, Su T-Z, Kocsis JD, Welty DF, Brown JP, Dooley DJ, Boden P, Singh L (1998). A summary of mechanistic hypotheses of gabapentin pharmacology. Epilepsy Res.

[58] Benelli R, Venè R, Ferrari N (2018). Prostaglandin-endoperoxide synthase 2 (cyclooxygenase-2), a complex target for colorectal cancer prevention and therapy. Transl Res.

[59] Alorabi M, Cavalu S, Al-kuraishy HM, Al-Gareeb AI, Mostafa-Hedeab G, Negm WA, Youssef A, El-Kadem AH, Saad HM, Batiha GE-S (2022). Pentoxifylline and berberine mitigate diclofenac-induced acute nephrotoxicity in male rats via modulation of inflammation and oxidative stress. Biomed Pharmacother.

[60] Grabauskas G, Wu X, Gao J, Li J-Y, Turgeon DK, Owyang C (2020). Prostaglandin E2, produced by mast cells in colon tissues from patients with irritable bowel syndrome, contributes to visceral hypersensitivity in mice. Gastroenterology.

[61] Laine L, Bombardier C, Hawkey CJ, Davis B, Shapiro D, Brett C, Reicin A (2002). Stratifying the risk of NSAID-related upper gastrointestinal clinical events: results of a double-blind outcomes study in patients with rheumatoid arthritis. Gastroenterology.

[62] Goto K, Ochi H, Yasunaga Y, Matsuyuki H, Imayoshi T, Kusuhara H, Okumoto T (1998). Analgesic effect of mofezolac, a non-steroidal anti-inflammatory drug, against phenylquinone-induced acute pain in mice. Prostaglandins Other Lipid Mediat.

[63] Manley PW, Allanson NM, Booth RF, Buckle PE, Kuzniar EJ, Lad N, Lai SM, Lunt DO, Tuffin DP (1987). Structure-activity relationships in an imidazole-based series of thromboxane synthase inhibitors. J Med Chem.

[64] Hu L, Yang Y, Tang Z, He Y, Luo X (2023). FCAN-MOPSO: an improved fuzzy-based graph clustering algorithm for complex networks with multi-objective particle swarm optimization. IEEE Trans Fuzzy Syst.

[65] Hu L, Zhang J, Pan X, Luo X, Yuan H (2021). An effective link-based clustering algorithm for detecting overlapping protein complexes in protein–protein interaction networks. IEEE Trans Netw Sci Eng.

[66] Rafiei F, Zeraati H, Abbasi K, Ghasemi JB, Parsaeian M, Masoudi-Nejad A (2023). DeepTraSynergy: drug combinations using multimodal deep learning with transformers. Bioinformatics.

