# Supplementary material for: Graph regularized non-negative matrix factorization with L2,1 norm regularization terms for drug–target interactions prediction
Source: BMC Bioinformatics. 2023 Oct 3;24:375. doi: 10.1186/s12859-023-05496-6 (PMC10548602; doi:10.1186/s12859-023-05496-6)
Supplement: Supplementary file 1 — Additional file 1. The supplementary material for iPALM-DLMF. [file 12859_2023_5496_MOESM1_ESM.pdf]

Graph regularized non-negative matrix factorization with $L_{2,1}$ norm regularization terms for drug-target interactions prediction(Appendix)

Junjun Zhang[1] and Minzhu Xie[1,2*]

[1] Key Laboratory of Computing and Stochastic Mathematics(LCSM) (Ministry of Education), School of Mathematics and Statistics, Hunan Normal University, Changsha 410081, China
[2] College of Information Science and Engineering, Hunan Normal University, Changsha 410081, China

Symbol meaning

$svd()$ represents singular value decomposition.
$\|\cdot\|$ represents norm.
$X(:,j)$ denotes the columns of X.
u^+ and v^+ denote the vector or matrix of same size that contains the same values as u and v, where u and v have nonnegative elements and 0 elsewhere.
u^- and v^- represent the vector or matrix of same size that contains the same values as u and v, where u and v have negative elements and 0 elsewhere.
$\|X^i\|_2$ represents the 2 norm of vector consisting of i-th row of X, $(i = 1, \cdots, n)$.
$\|Y^j\|_2$ represents the 2 norm of vector consisting of j-th row of Y, $(j = 1, \cdots, m)$.

The detailed steps of NNDSVD

Table S1: NNDSVD

Inputs: Matrix $Z \in R^{n\times m}$, integer $k < min(m,n)$.
Output: Rank-k nonnegative factors $X \in R^{n\times k}, B \in Y^{m\times k}$.
1. Compute the largest k singular triplets of Z : $[U,S,V] = svd(Z,k)$
2. Initialize $X(:,1) = \sqrt{S(1,1)} * U(:,1)$ and $Y(:,1) = \sqrt{S(1,1)} * V(:,1)$
3. for $j = 2 : k$
3.1. $u = U(:,j)$; $v = V(:,j)$;
3.2. $up = u^+$; $un = u^-$; $vp = v^+$; $vn = v^-$;
3.3. $upnrm = \|up\|$; $vpnrm = \|vp\|$; $mp = upnrm * vpnrm$;
3.5. $unnrm = \|un\|$; $vnnrm = \|vn\|$; $mn = unnrm * vnnrm$;
3.6. if $mp > mn$:
$p = up/upnrm$; $q = vp/vpnrm$;$\sigma = mp$;
else
$p = un/unnrm$; $q = vn/ynnrm$; $\sigma = mn$;
endif
3.7. $X(:,j) = \sqrt{S(j,j) * \sigma} * p$ and $Y(:,j) = \sqrt{S(j,j) * \sigma} * q$;

The derivation of formula 17

In order to prove that the formula (17) is a solution of model (10), we adopt the proof by contradiction, and the proof process is as follows:

$$X^{i+1} \in prox_{c_1^i}^{\delta_X}(X^i - \frac{1}{c_1^i}\nabla_X G(X^i, Y^i)) \tag{1}$$

$$= \arg\min_X(\delta_X + \frac{1}{2\frac{1}{c_1^i}}\left\|u - (X^i - \frac{1}{c_1^i}\nabla_X G(X^i, Y^i))\right\|_F^2) \tag{2}$$

$$= \arg\min_X(\delta_X + \frac{c_1^i}{2}\left\|u - X^i + \frac{1}{c_1^i}\nabla_X G(X^i, Y^i))\right\|_F^2) \tag{3}$$

$$= \arg\min_X(\delta_X + \frac{1}{2c_1^i}\left\|\nabla_X G(X^i, Y^i))\right\|_F^2 + \nabla_X G(X^i, Y^i)(u - X^i) + (\frac{c_1^i}{2}\left\|u - X^i\right\|_F^2)) \tag{4}$$

$$= \arg\min_X(\delta_X + G(X^i, Y^i) + \nabla_X G(X^i, Y^i)(u - X^i) + \frac{c_1^i}{2}\left\|u - X^i\right\|_F^2). \tag{5}$$

The formula (4) can be obtained by expanding the square term of the formula (3). Since there is no variable u in $\frac{1}{2c_1^i}\left\|\nabla_X G(X^i, Y^i))\right\|_F^2$ of formula (4), $\frac{1}{2c_1^i}\left\|\nabla_X G(X^i, Y^i))\right\|_F^2$ is regarded as constant term in the formula (5), then the solution of formula (5) is the solution of formula (16).
