# Supplementary material for: Graph regularized non-negative matrix factorization with L2,1 norm regularization terms for drug–target interactions prediction
Source: BMC Bioinformatics. 2023 Oct 3;24:375. doi: 10.1186/s12859-023-05496-6 (PMC10548602; doi:10.1186/s12859-023-05496-6)
Supplement: Supplementary file 2 — Additional file 2. iPALM-DLMF + appendix. [file 12859_2023_5496_MOESM2_ESM.zip › iPALM-DLMF/iPframe.pdf]

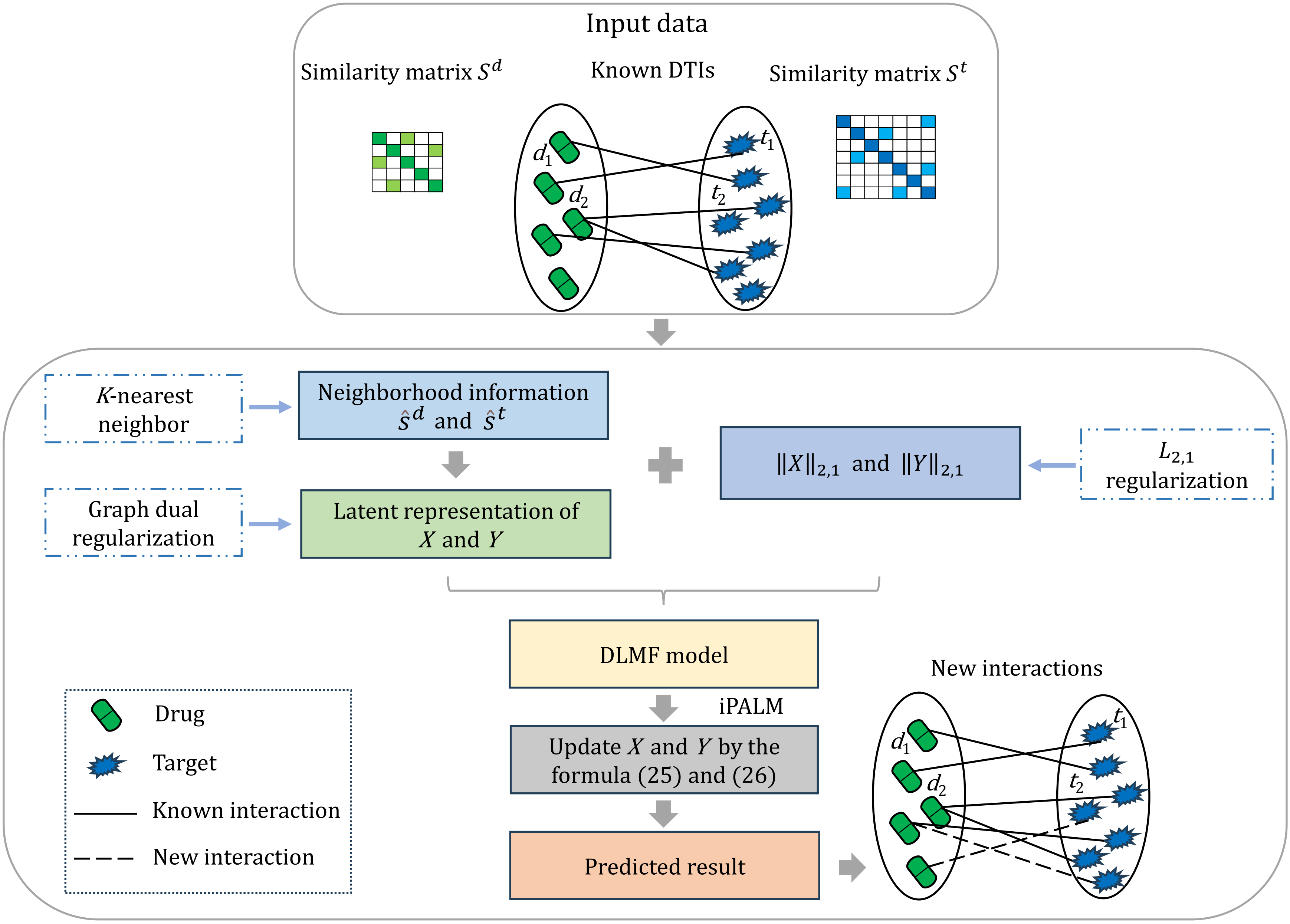

Input data
Similarity matrix S^d
Known DTIs
Similarity matrix S^t
d_1
d_2
t_1
t_2
K-nearest neighbor
Neighborhood information $\hat{s}^d$ and $\hat{s}^t$
Graph dual regularization
Latent representation of X and Y
$\|X\|_{2,1}$ and $\|Y\|_{2,1}$
$L_{2,1}$ regularization
DLMF model
iPALM
Update X and Y by the formula (25) and (26)
Predicted result
New interactions
Drug
Target
Known interaction
New interaction
