# Supplementary figures and images for: Graph regularized non-negative matrix factorization with L2,1 norm regularization terms for drug–target interactions prediction
Source: BMC Bioinformatics. 2023 Oct 3;24:375. doi: 10.1186/s12859-023-05496-6 (PMC10548602; doi:10.1186/s12859-023-05496-6)

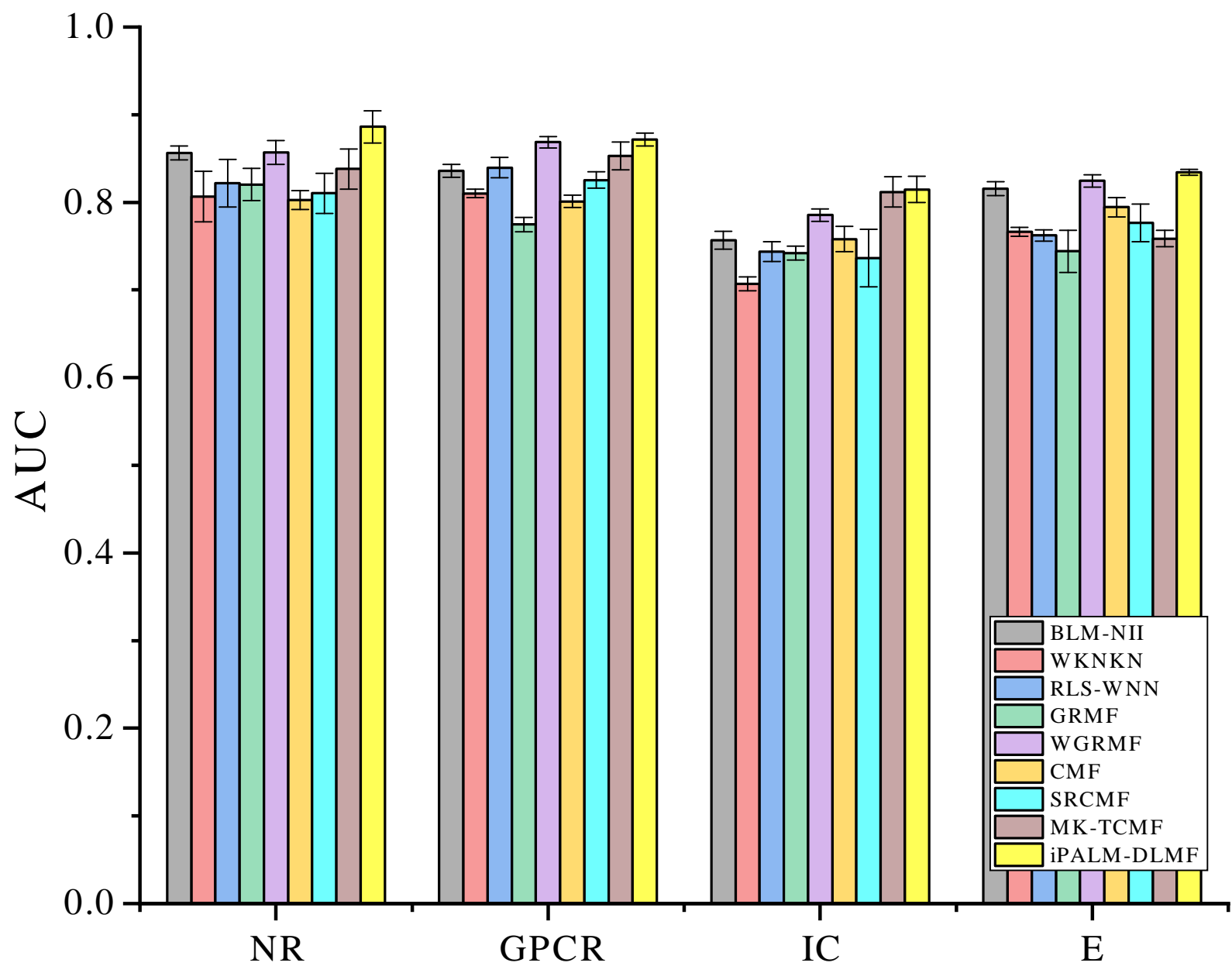

Supplement: Supplementary file 2 — Additional file 2. iPALM-DLMF + appendix. [file 12859_2023_5496_MOESM2_ESM.zip › iPALM-DLMF/auccvd.pdf]

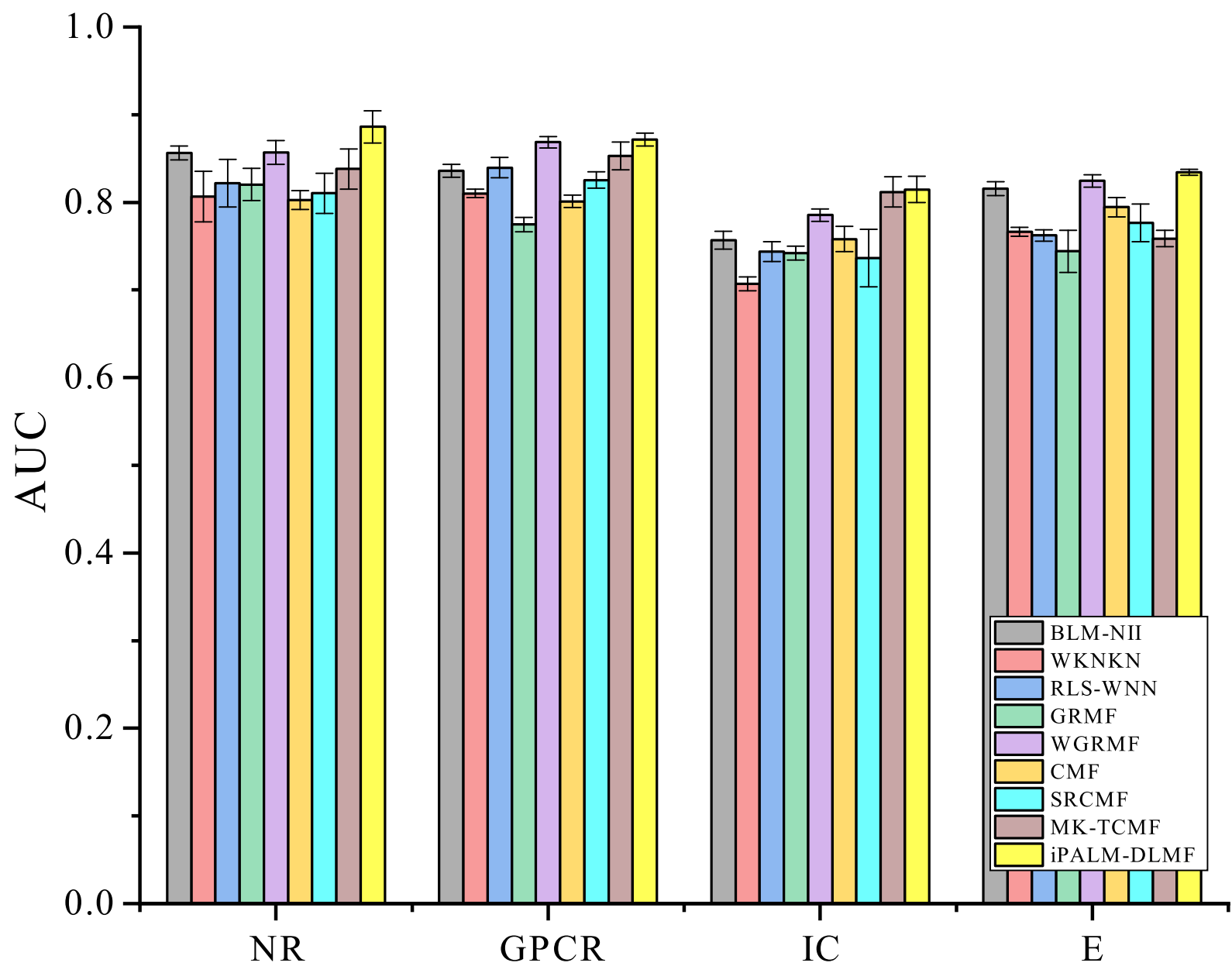

Supplement: Supplementary file 2 — Additional file 2. iPALM-DLMF + appendix. [file 12859_2023_5496_MOESM2_ESM.zip › iPALM-DLMF/auccvd-eps-converted-to.pdf]

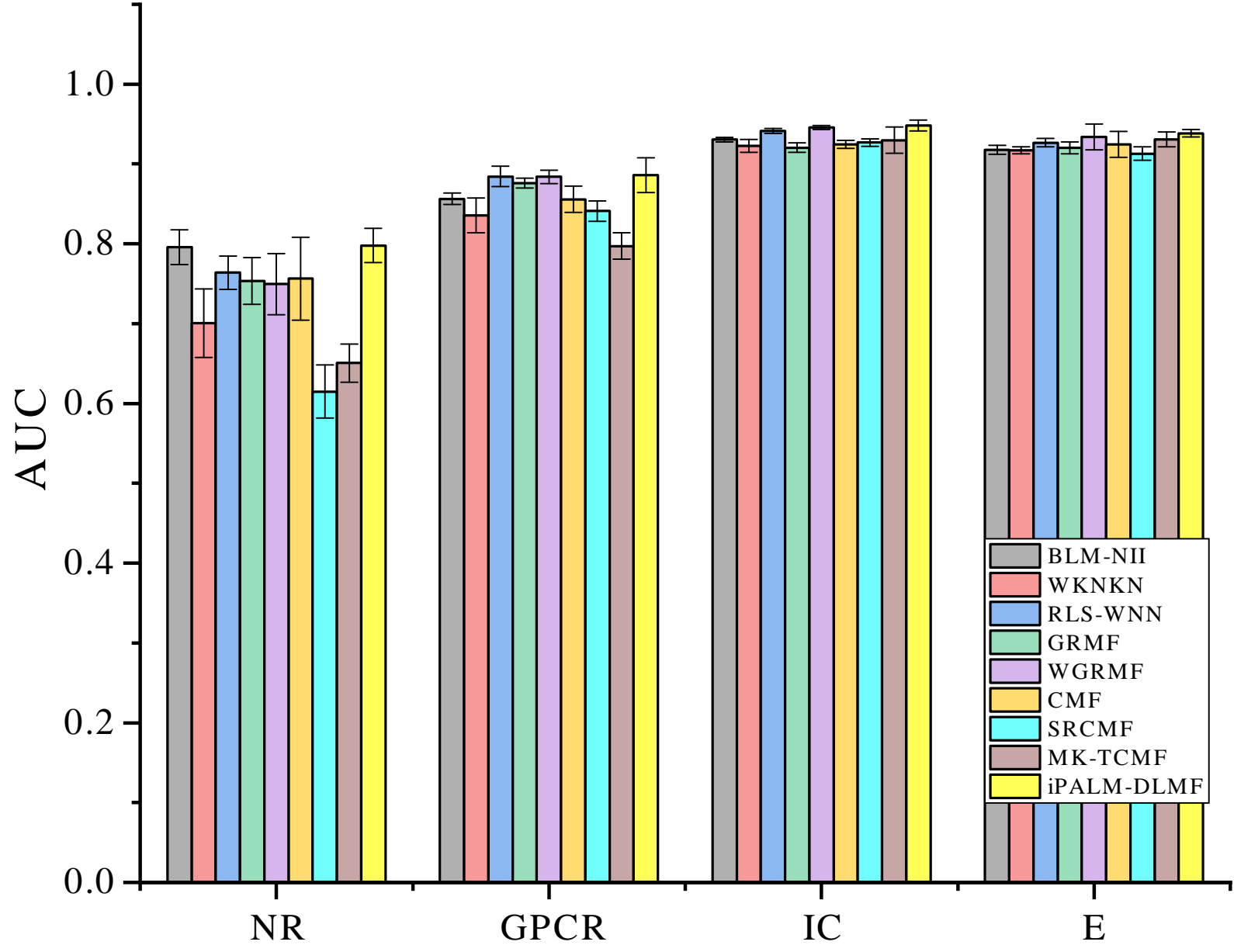

Supplement: Supplementary file 2 — Additional file 2. iPALM-DLMF + appendix. [file 12859_2023_5496_MOESM2_ESM.zip › iPALM-DLMF/auccvt.pdf]

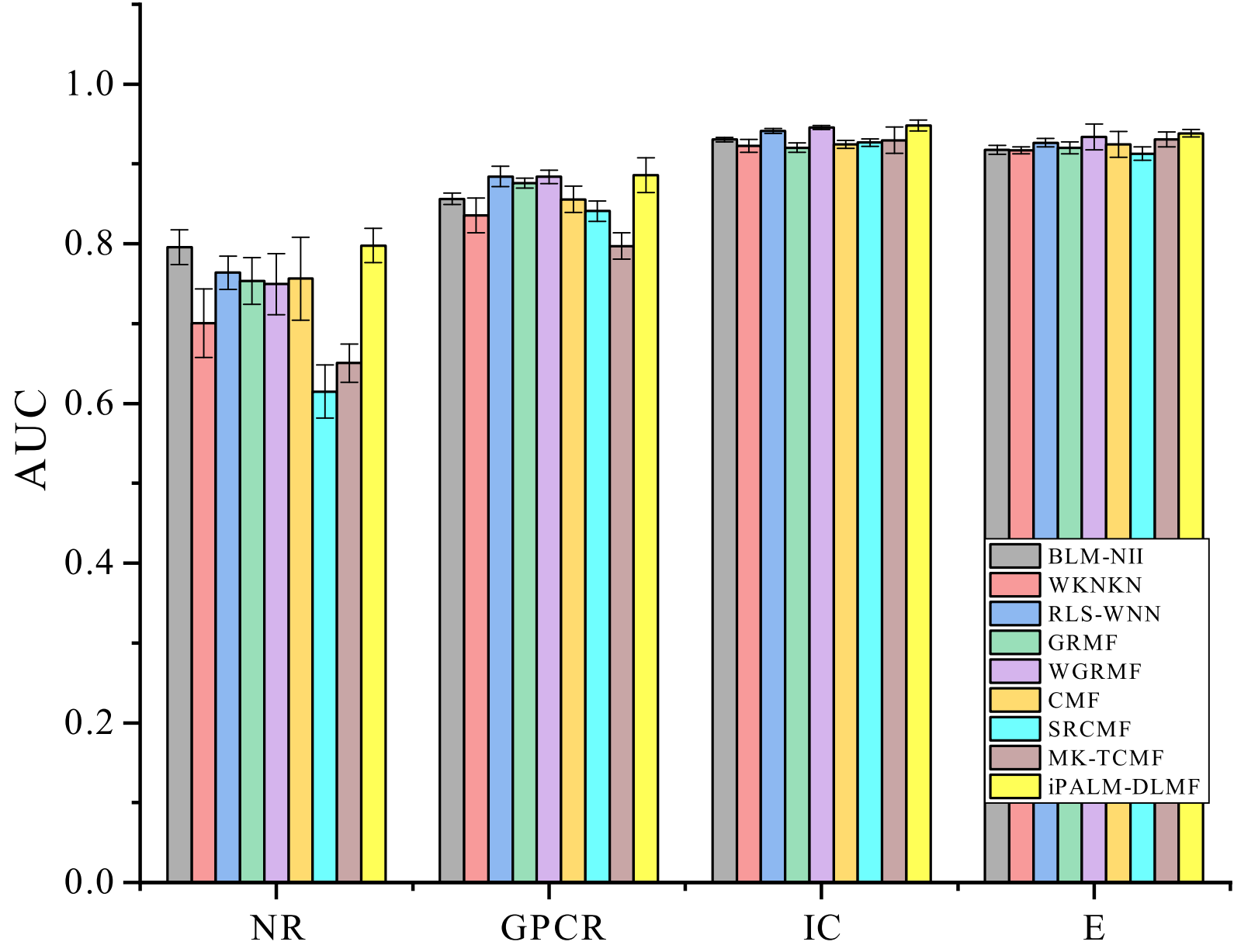

Supplement: Supplementary file 2 — Additional file 2. iPALM-DLMF + appendix. [file 12859_2023_5496_MOESM2_ESM.zip › iPALM-DLMF/auccvt-eps-converted-to.pdf]

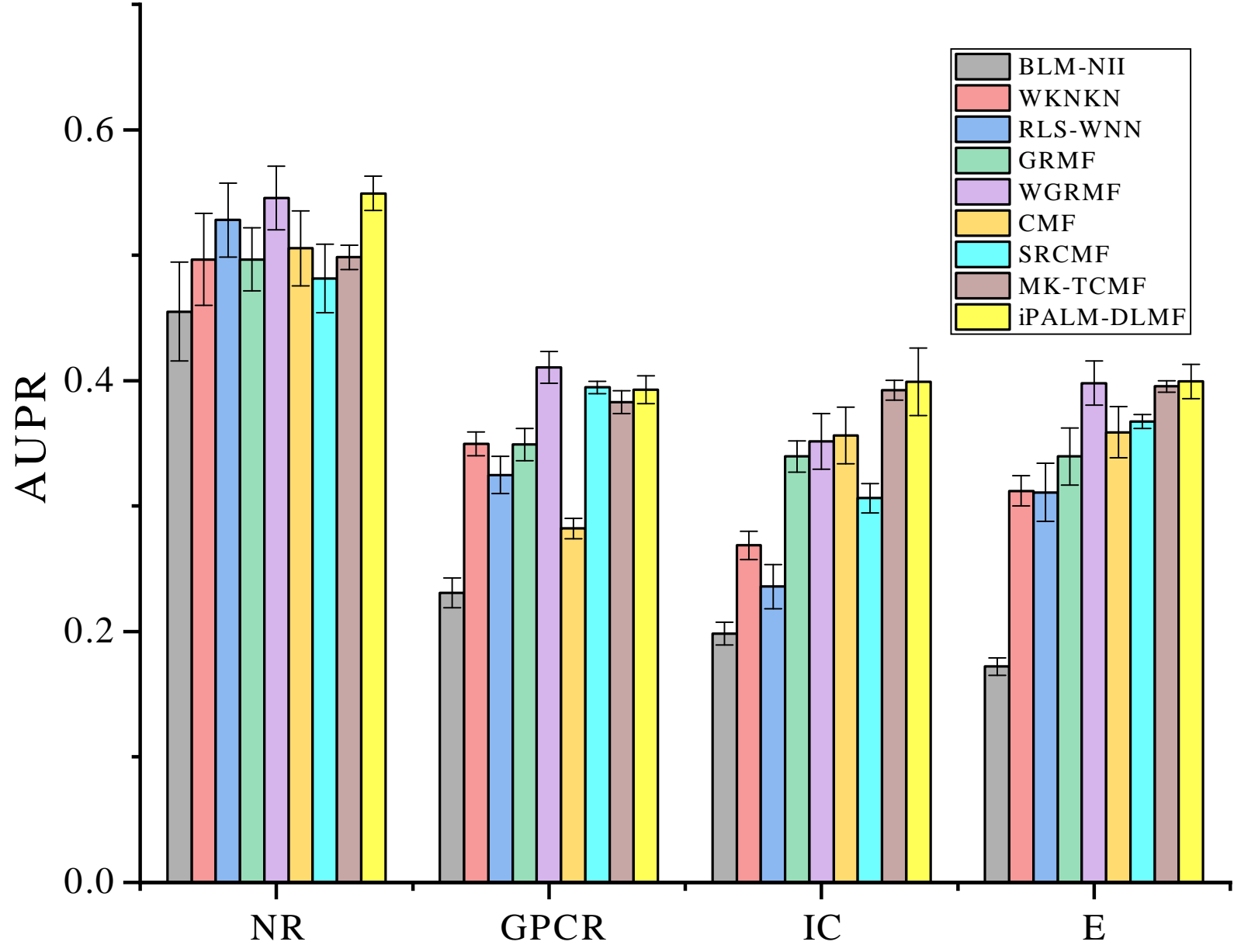

Supplement: Supplementary file 2 — Additional file 2. iPALM-DLMF + appendix. [file 12859_2023_5496_MOESM2_ESM.zip › iPALM-DLMF/auprcvd.pdf]

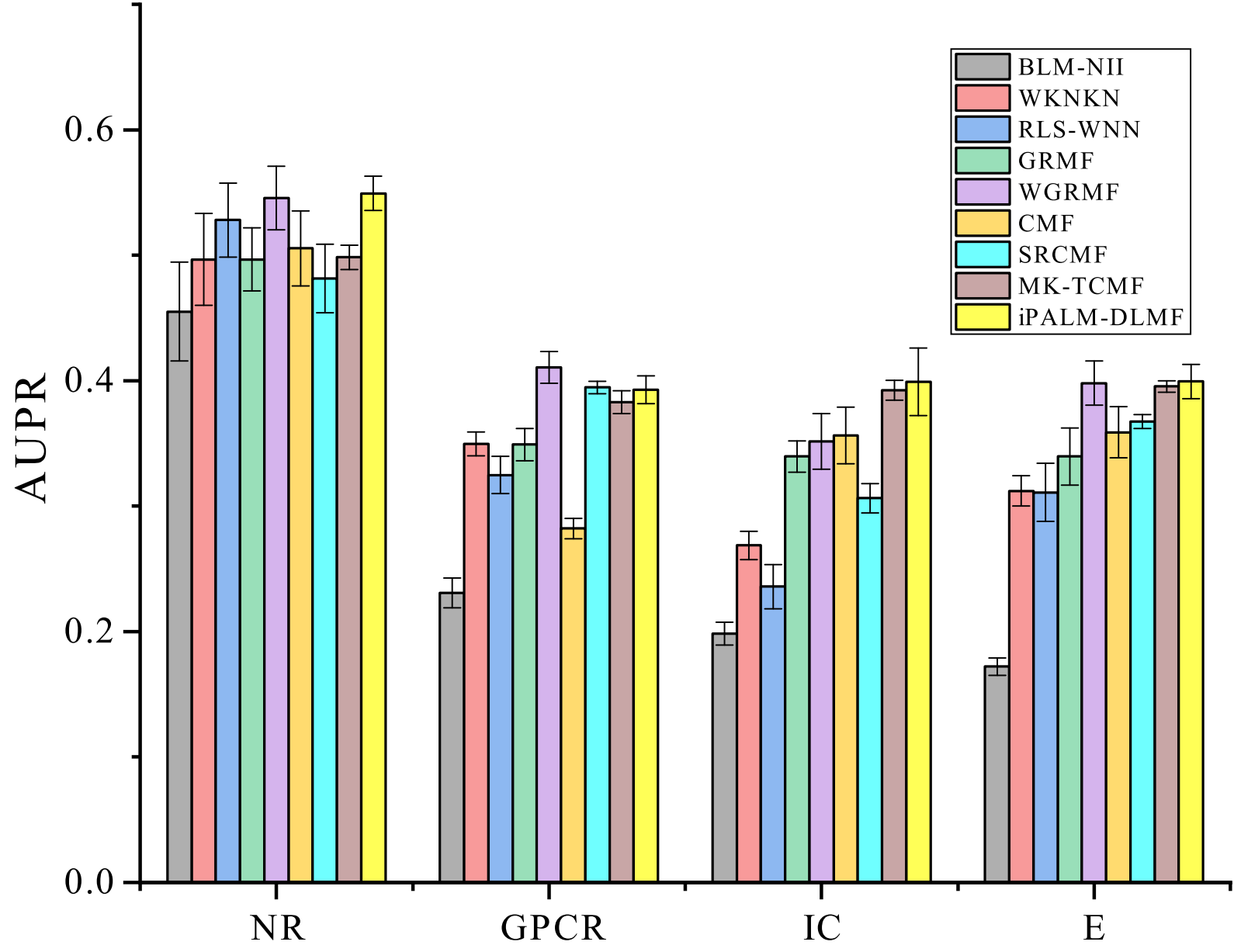

Supplement: Supplementary file 2 — Additional file 2. iPALM-DLMF + appendix. [file 12859_2023_5496_MOESM2_ESM.zip › iPALM-DLMF/auprcvd-eps-converted-to.pdf]

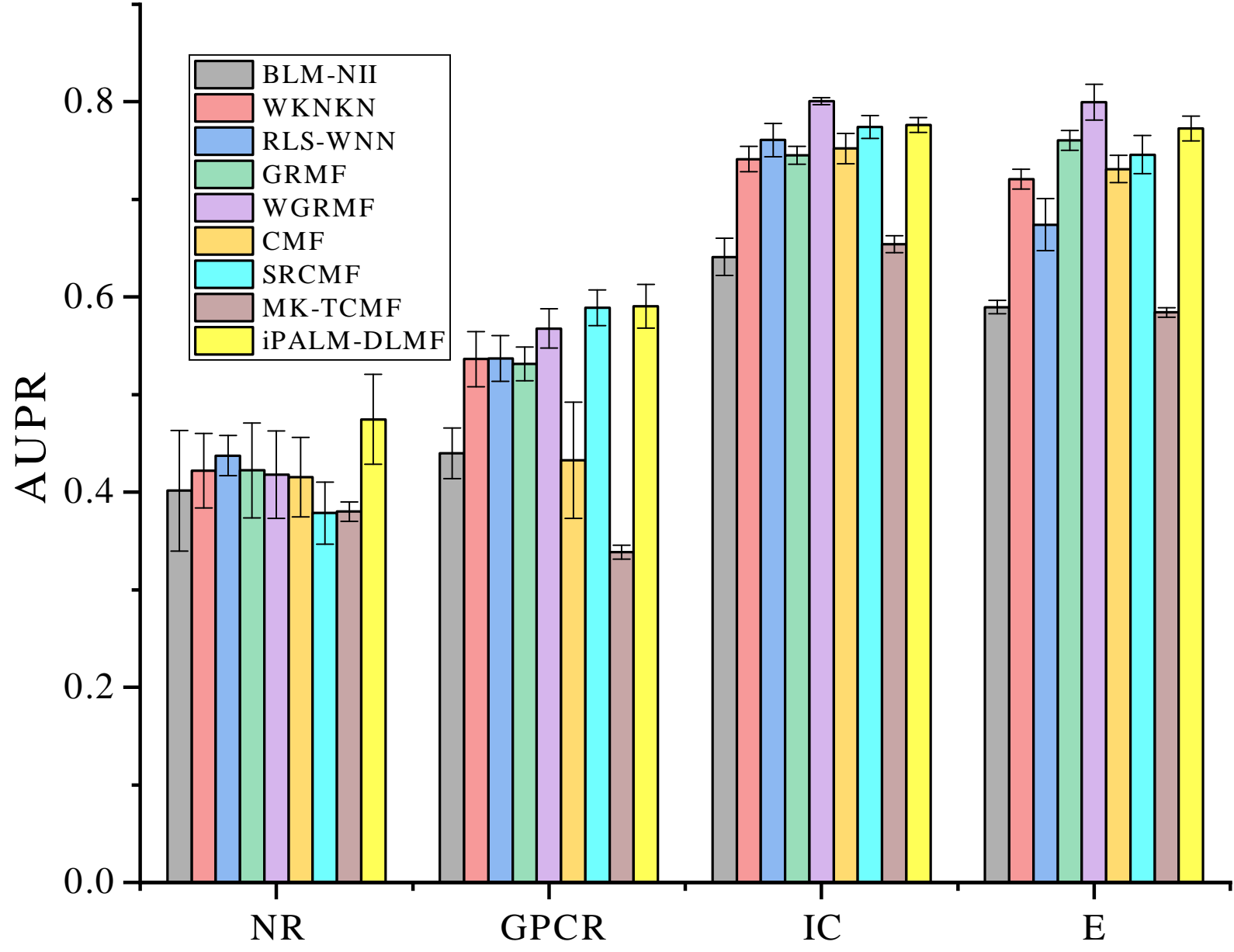

Supplement: Supplementary file 2 — Additional file 2. iPALM-DLMF + appendix. [file 12859_2023_5496_MOESM2_ESM.zip › iPALM-DLMF/auprcvt.pdf]

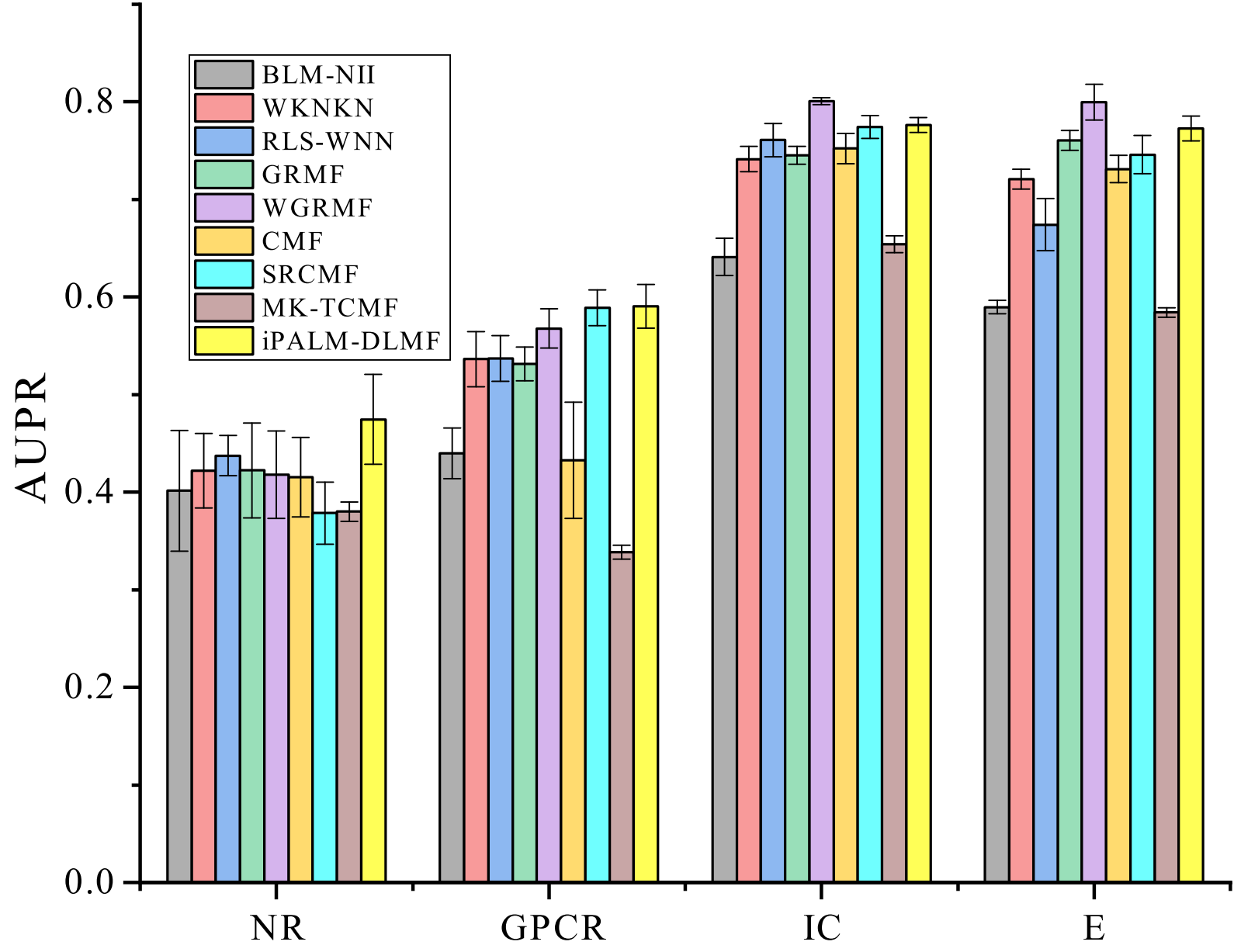

Supplement: Supplementary file 2 — Additional file 2. iPALM-DLMF + appendix. [file 12859_2023_5496_MOESM2_ESM.zip › iPALM-DLMF/auprcvt-eps-converted-to.pdf]

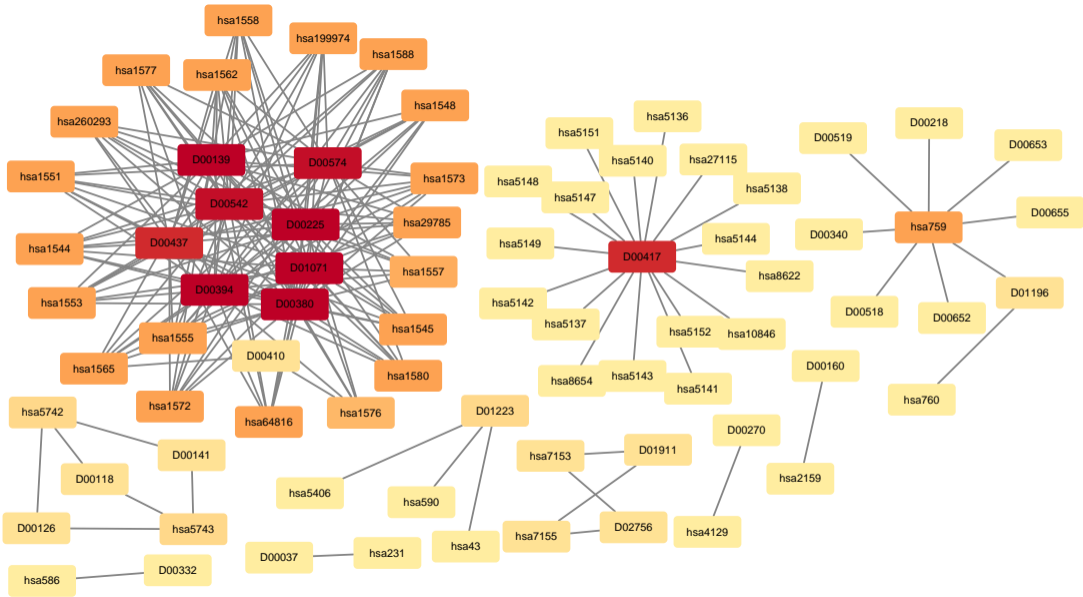

Supplement: Supplementary file 2 — Additional file 2. iPALM-DLMF + appendix. [file 12859_2023_5496_MOESM2_ESM.zip › iPALM-DLMF/IPnetwork.pdf]

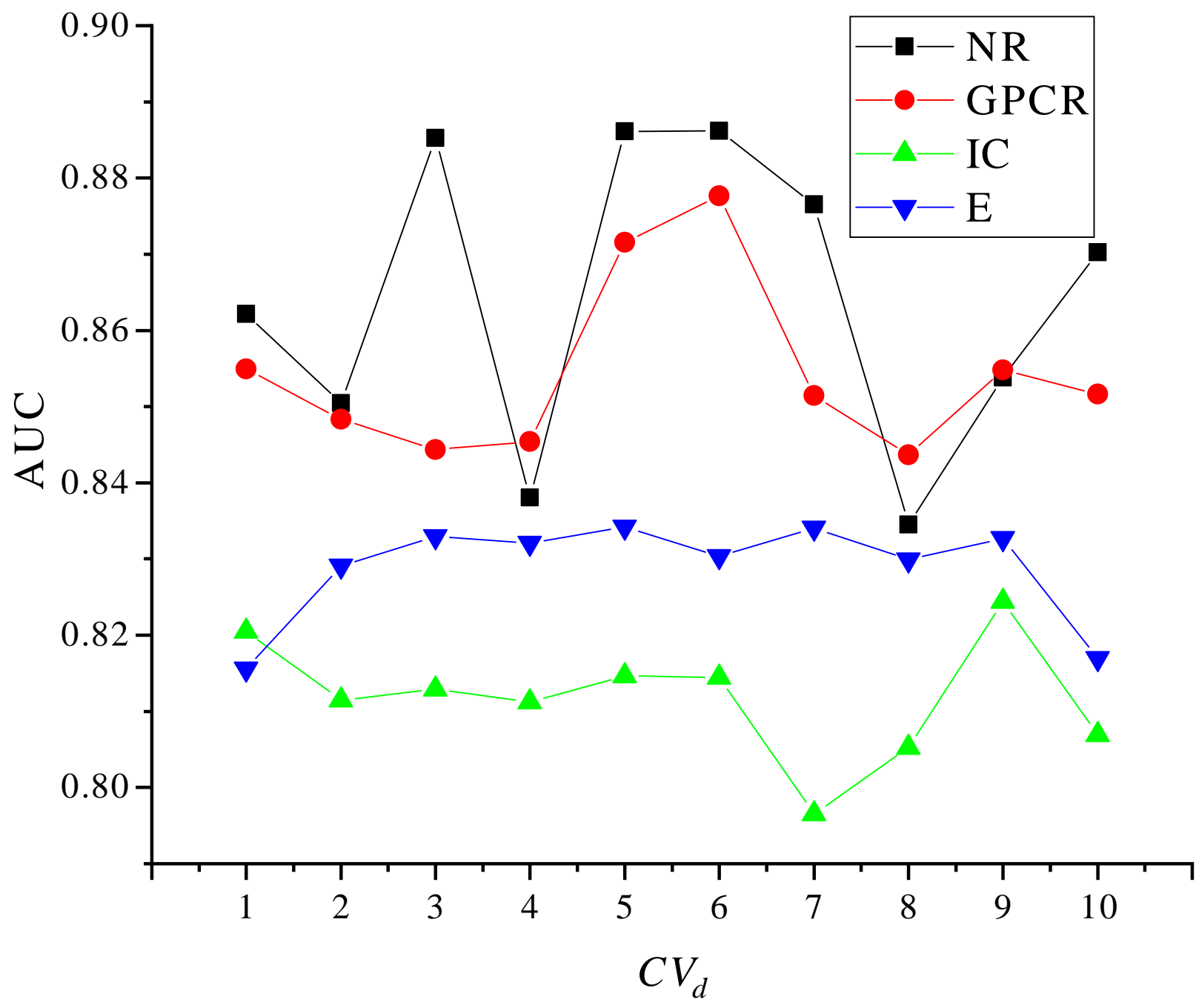

Supplement: Supplementary file 2 — Additional file 2. iPALM-DLMF + appendix. [file 12859_2023_5496_MOESM2_ESM.zip › iPALM-DLMF/kauccvd.pdf]

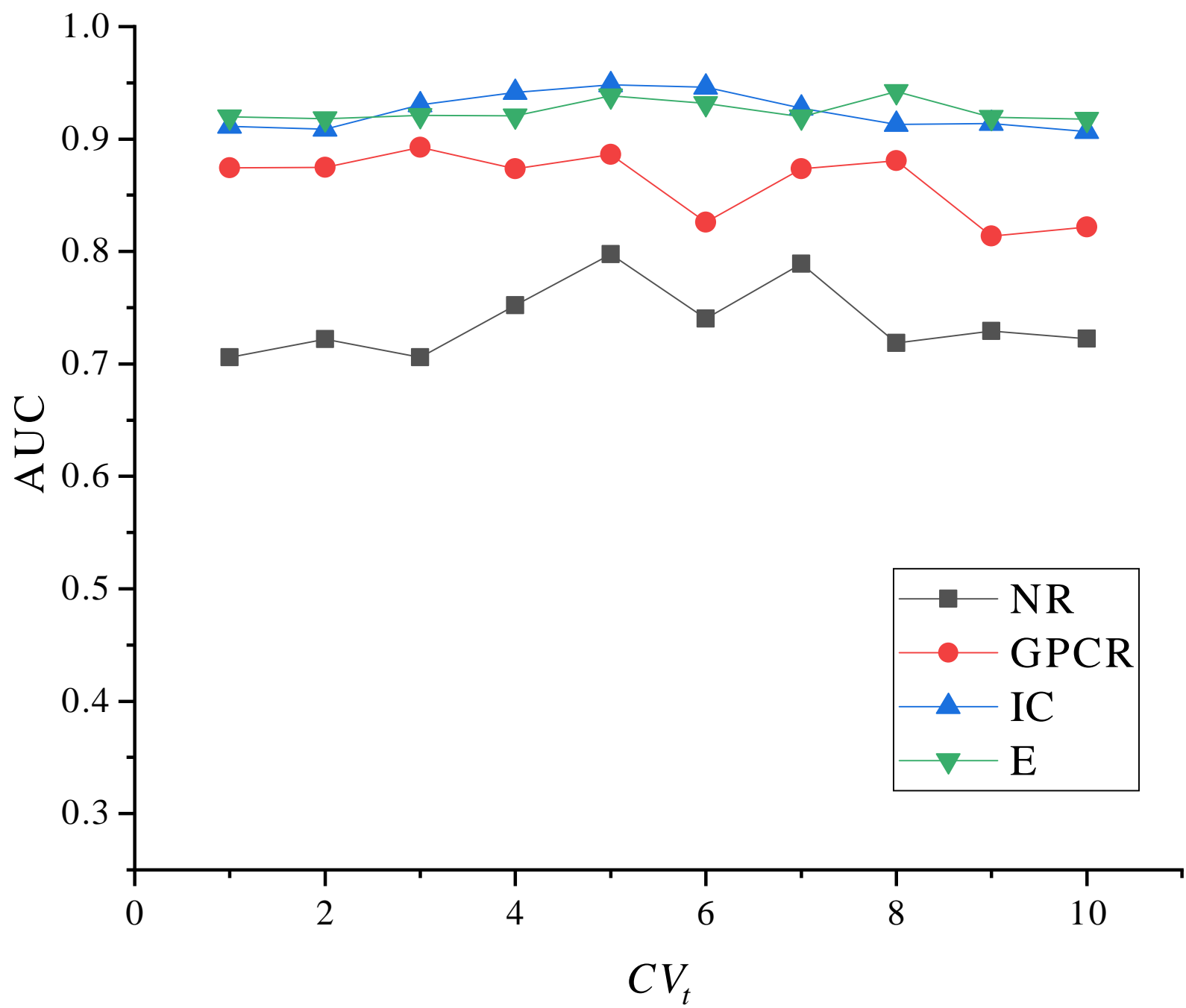

Supplement: Supplementary file 2 — Additional file 2. iPALM-DLMF + appendix. [file 12859_2023_5496_MOESM2_ESM.zip › iPALM-DLMF/kauccvt.pdf]

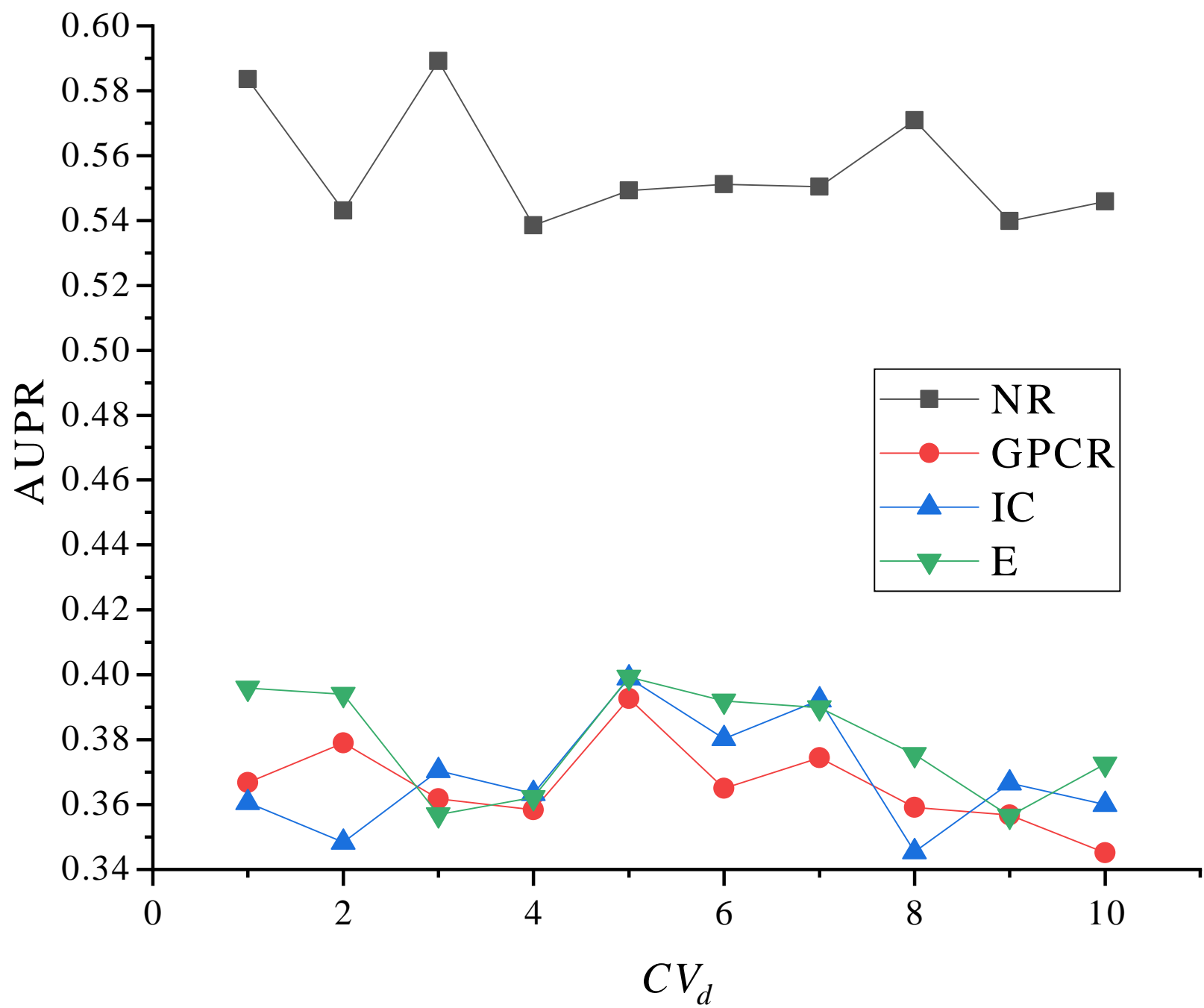

Supplement: Supplementary file 2 — Additional file 2. iPALM-DLMF + appendix. [file 12859_2023_5496_MOESM2_ESM.zip › iPALM-DLMF/kauprcvd.pdf]

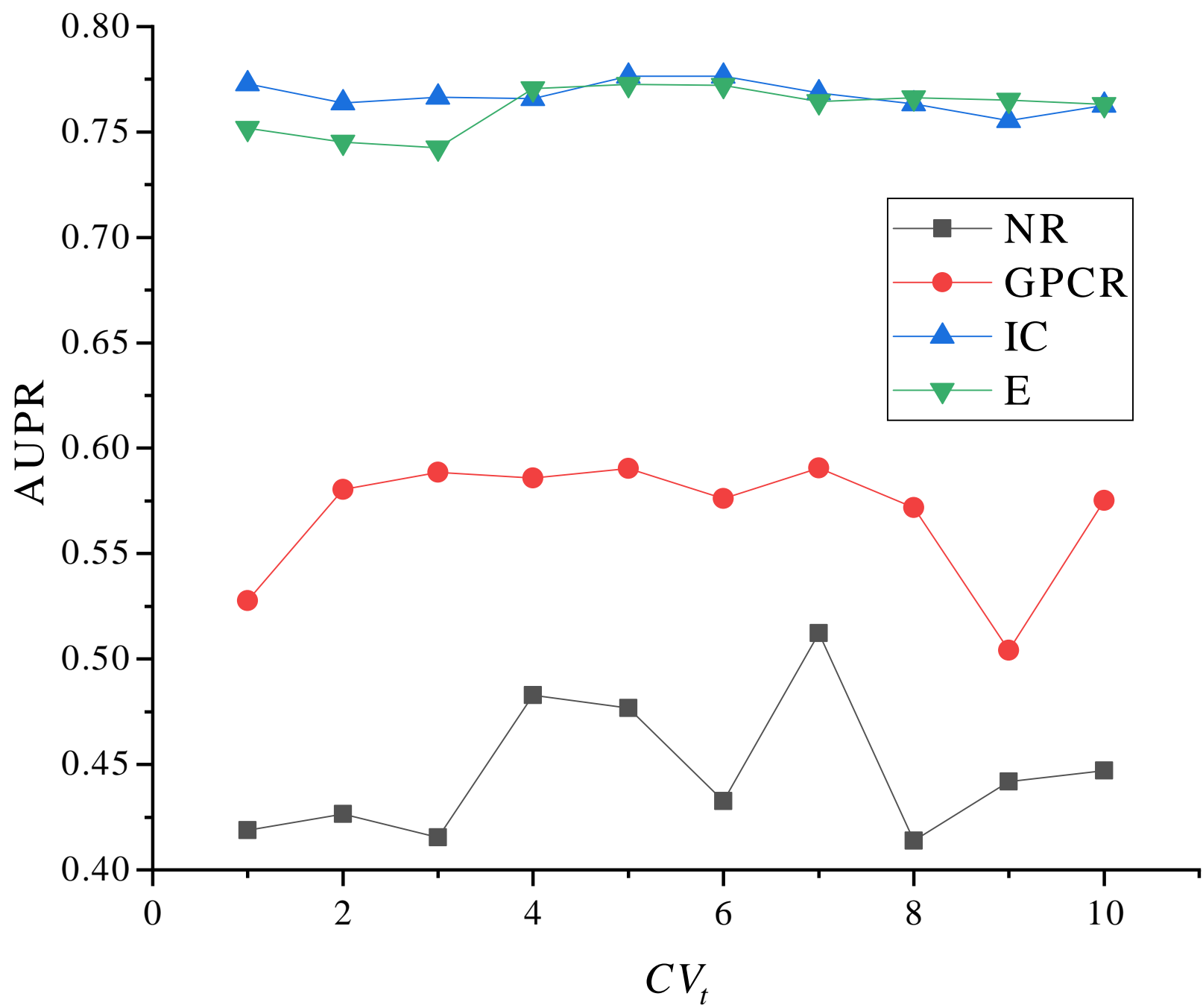

Supplement: Supplementary file 2 — Additional file 2. iPALM-DLMF + appendix. [file 12859_2023_5496_MOESM2_ESM.zip › iPALM-DLMF/kauprcvt.pdf]

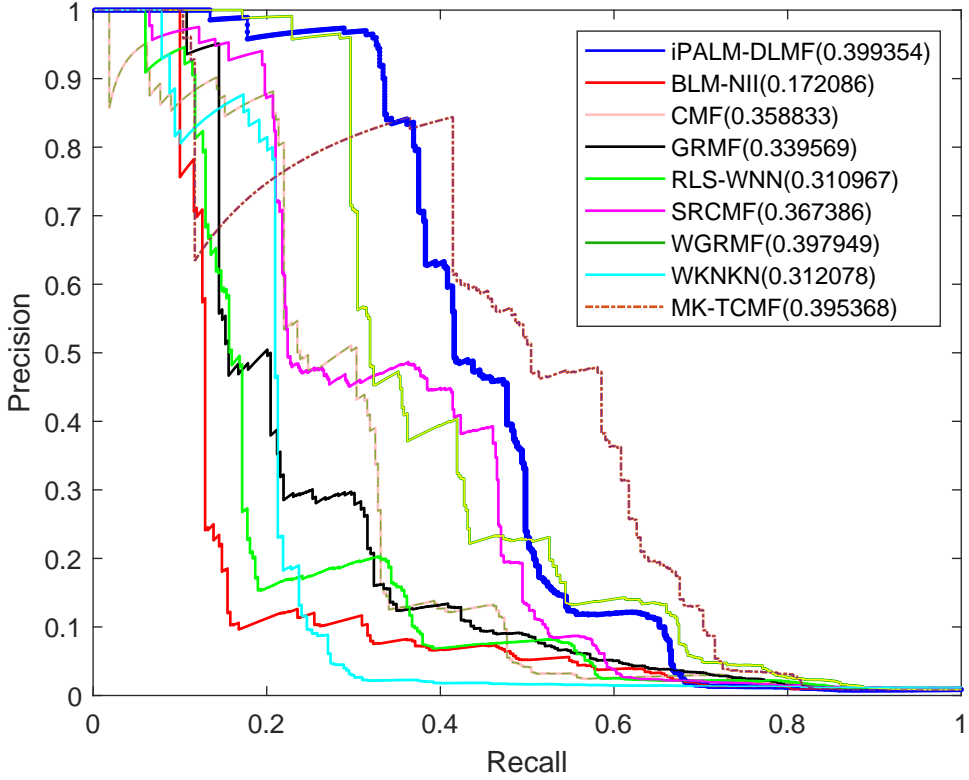

Supplement: Supplementary file 2 — Additional file 2. iPALM-DLMF + appendix. [file 12859_2023_5496_MOESM2_ESM.zip › iPALM-DLMF/precvd.pdf]

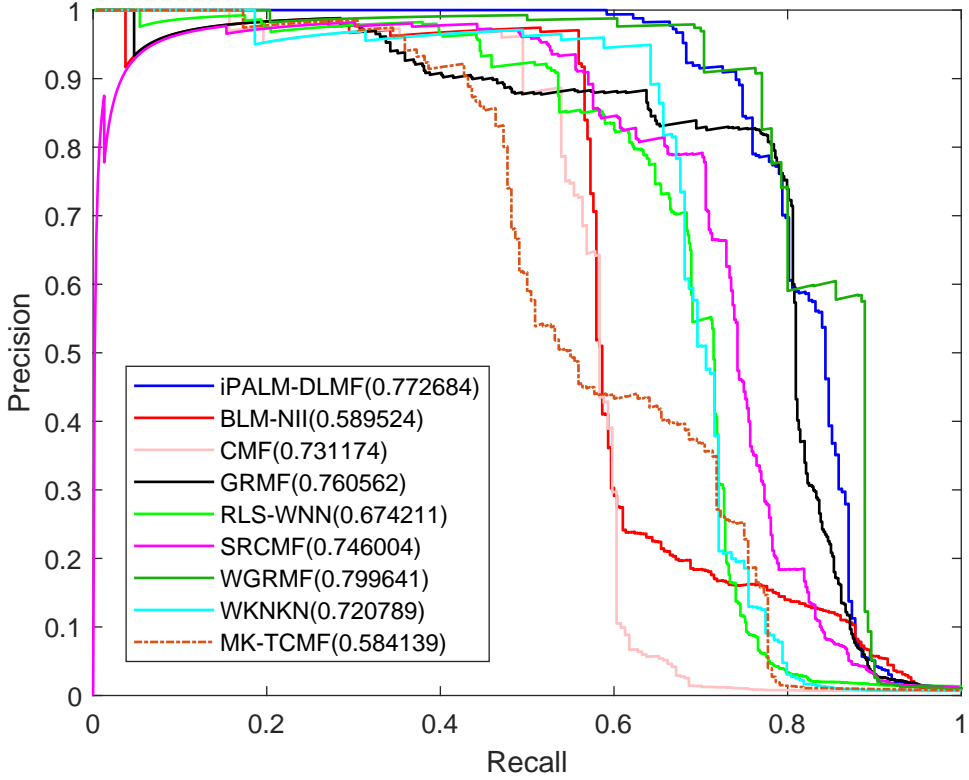

Supplement: Supplementary file 2 — Additional file 2. iPALM-DLMF + appendix. [file 12859_2023_5496_MOESM2_ESM.zip › iPALM-DLMF/precvt.pdf]

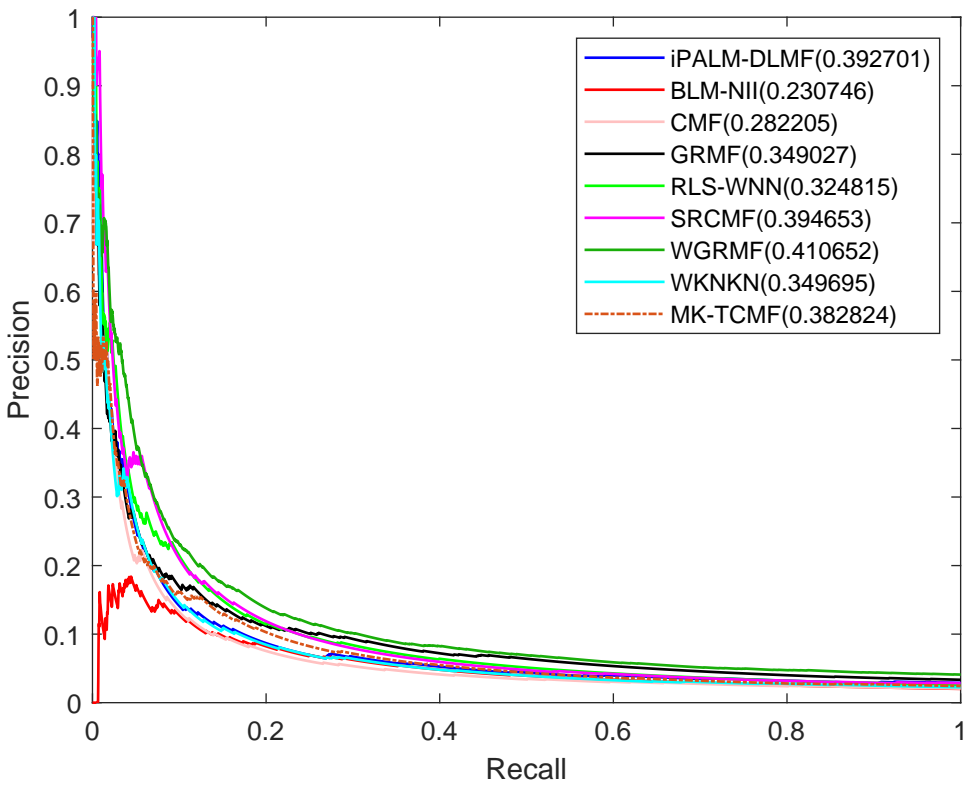

Supplement: Supplementary file 2 — Additional file 2. iPALM-DLMF + appendix. [file 12859_2023_5496_MOESM2_ESM.zip › iPALM-DLMF/prgpcrcvd.pdf]

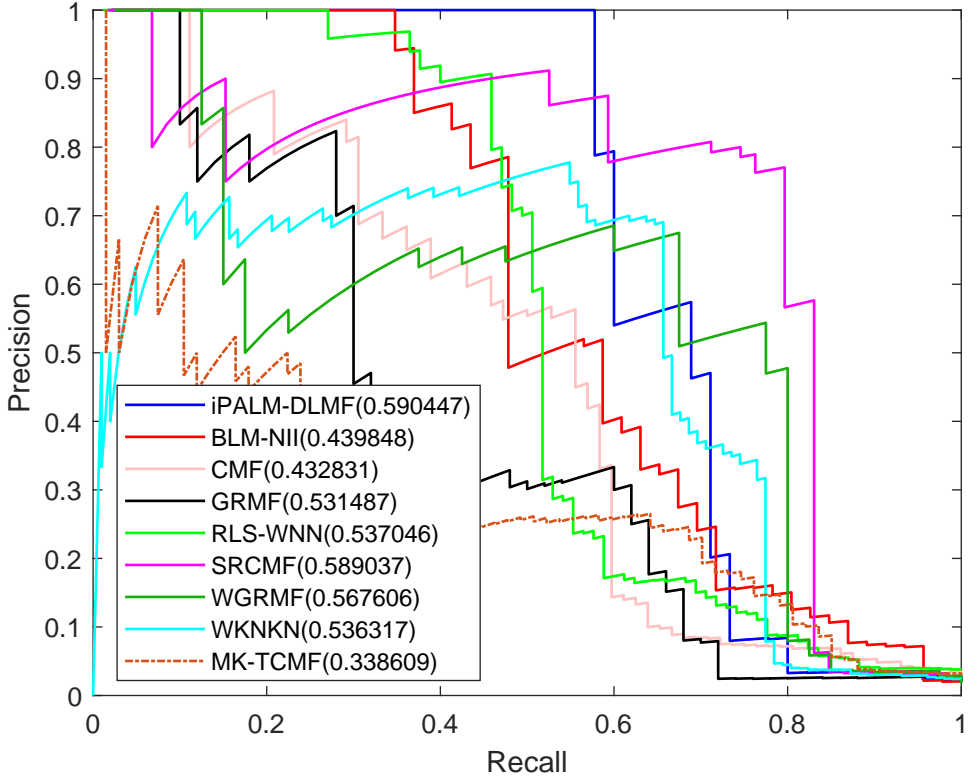

Supplement: Supplementary file 2 — Additional file 2. iPALM-DLMF + appendix. [file 12859_2023_5496_MOESM2_ESM.zip › iPALM-DLMF/prgpcrcvt.pdf]

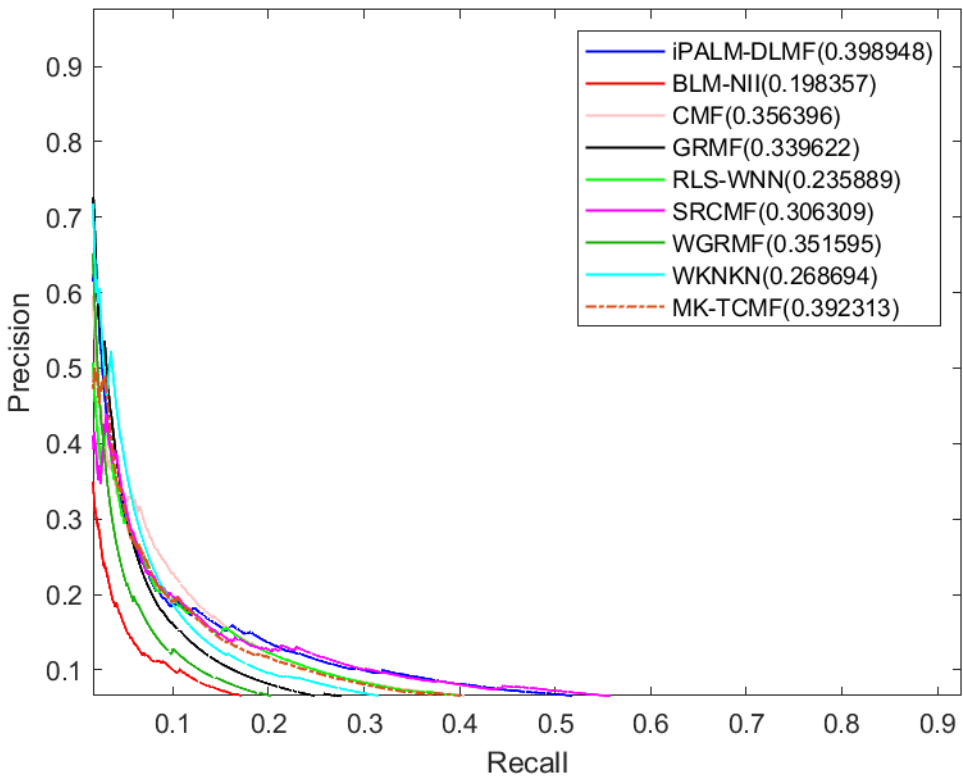

Supplement: Supplementary file 2 — Additional file 2. iPALM-DLMF + appendix. [file 12859_2023_5496_MOESM2_ESM.zip › iPALM-DLMF/priccvd.pdf]

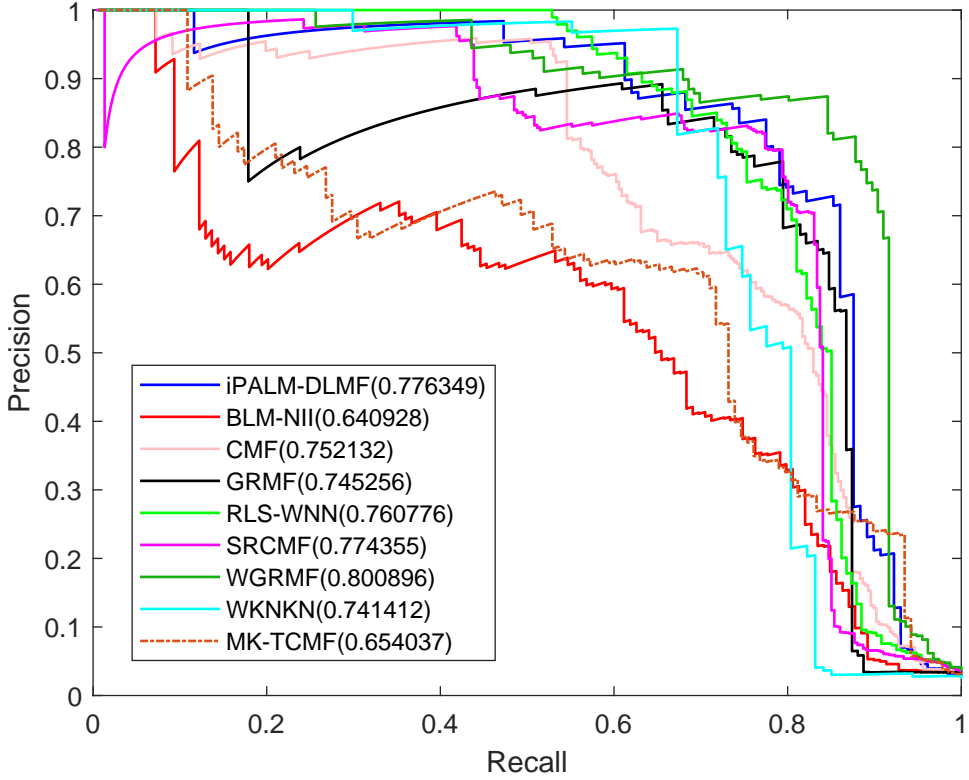

Supplement: Supplementary file 2 — Additional file 2. iPALM-DLMF + appendix. [file 12859_2023_5496_MOESM2_ESM.zip › iPALM-DLMF/priccvt.pdf]

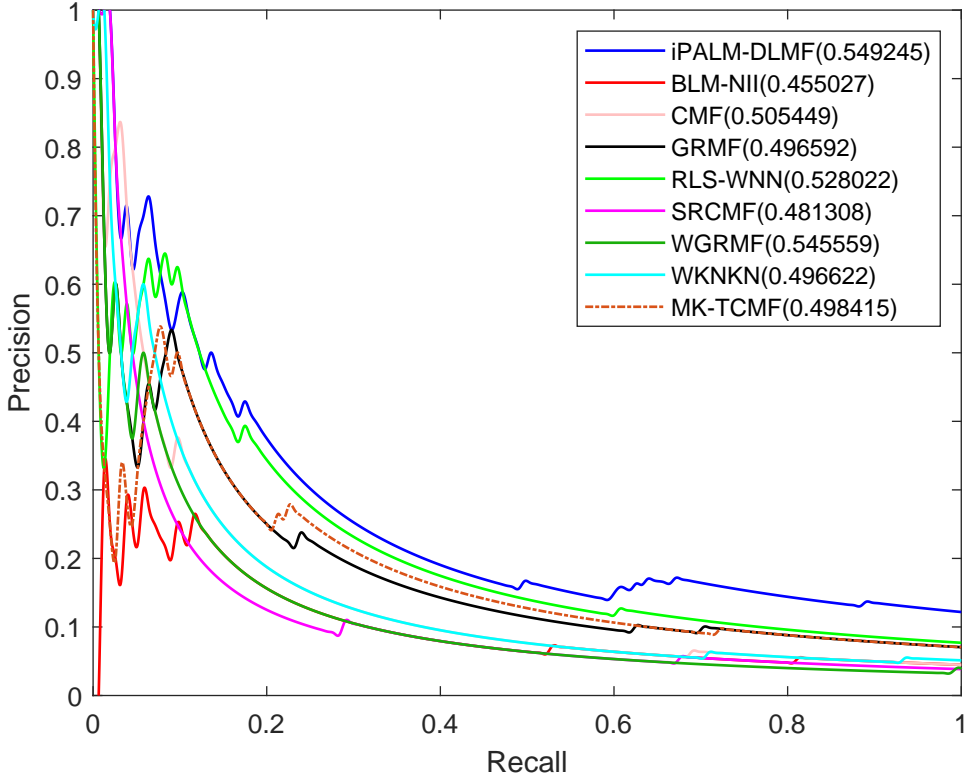

Supplement: Supplementary file 2 — Additional file 2. iPALM-DLMF + appendix. [file 12859_2023_5496_MOESM2_ESM.zip › iPALM-DLMF/prnrcvd.pdf]

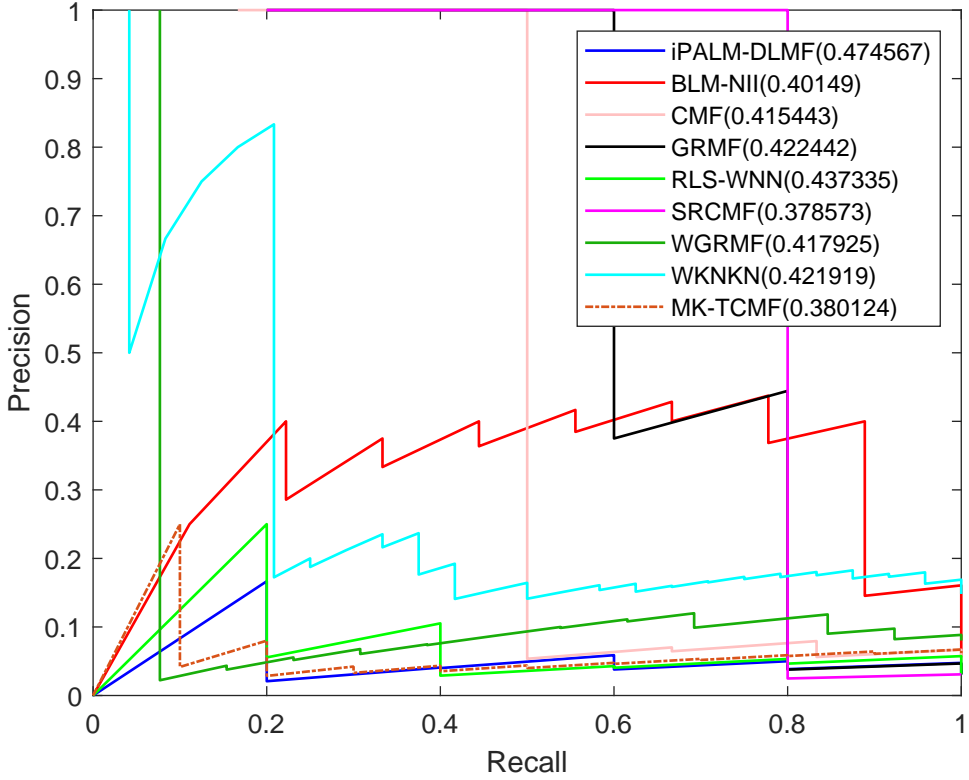

Supplement: Supplementary file 2 — Additional file 2. iPALM-DLMF + appendix. [file 12859_2023_5496_MOESM2_ESM.zip › iPALM-DLMF/prnrcvt.pdf]

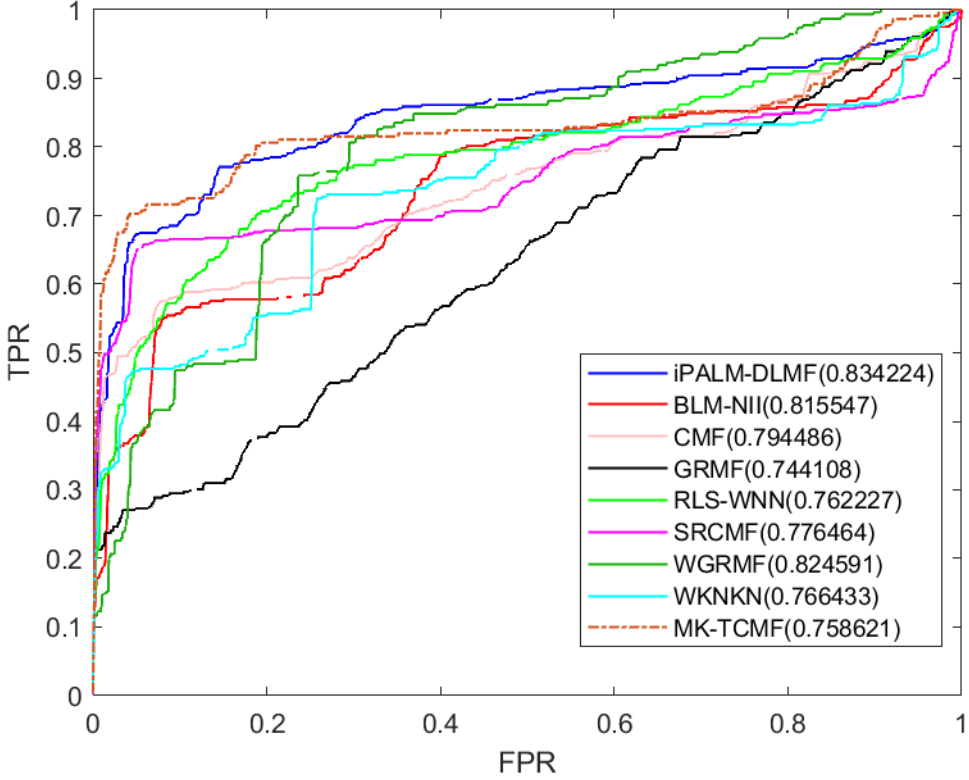

Supplement: Supplementary file 2 — Additional file 2. iPALM-DLMF + appendix. [file 12859_2023_5496_MOESM2_ESM.zip › iPALM-DLMF/rocecvd.pdf]

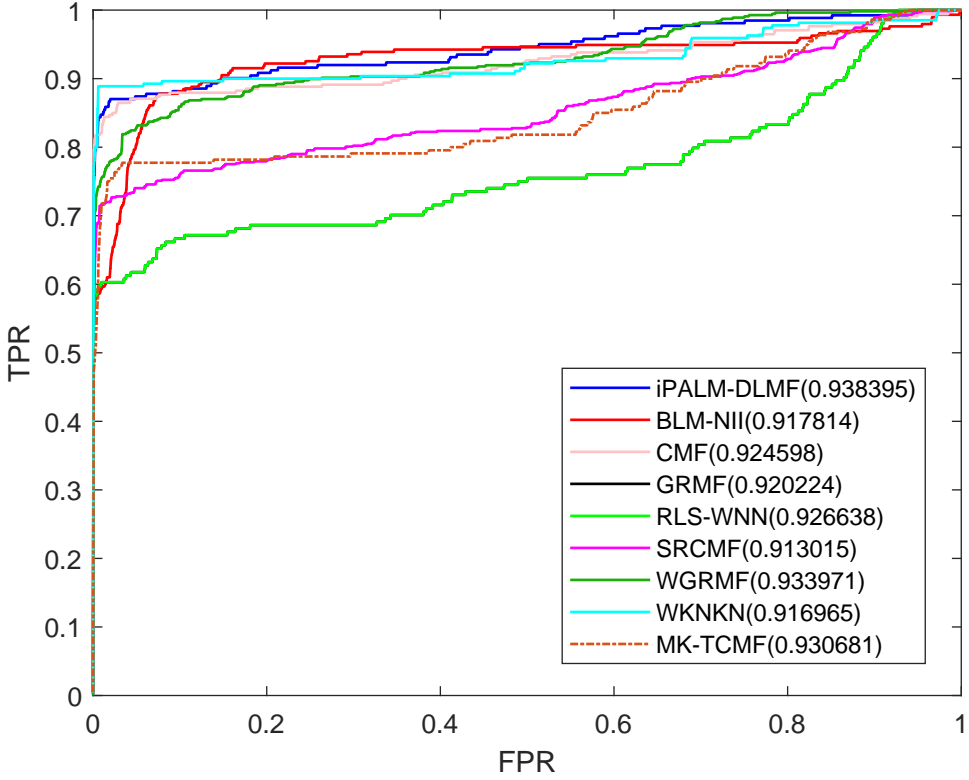

Supplement: Supplementary file 2 — Additional file 2. iPALM-DLMF + appendix. [file 12859_2023_5496_MOESM2_ESM.zip › iPALM-DLMF/rocecvt.pdf]

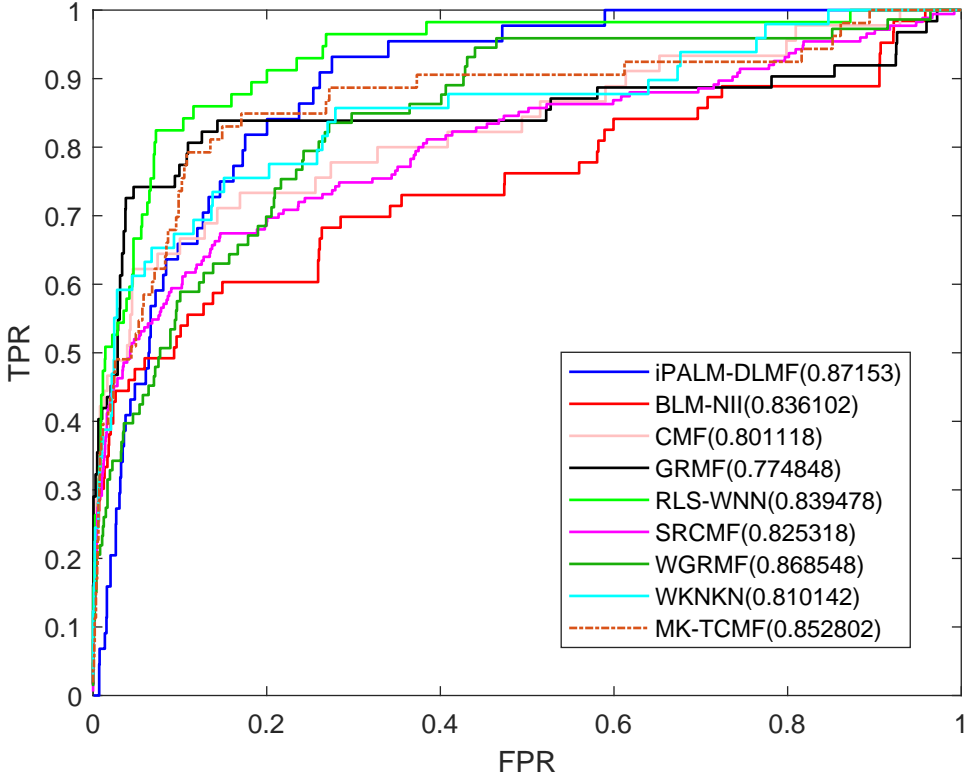

Supplement: Supplementary file 2 — Additional file 2. iPALM-DLMF + appendix. [file 12859_2023_5496_MOESM2_ESM.zip › iPALM-DLMF/rocgpcrcvd.pdf]

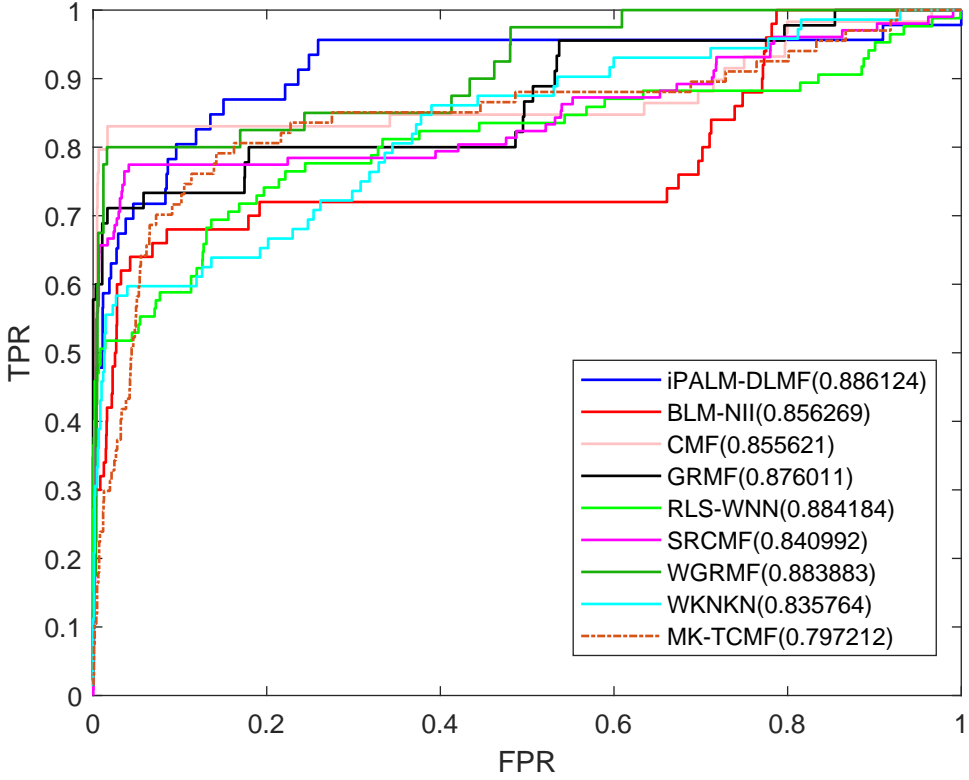

Supplement: Supplementary file 2 — Additional file 2. iPALM-DLMF + appendix. [file 12859_2023_5496_MOESM2_ESM.zip › iPALM-DLMF/rocgpcrcvt.pdf]

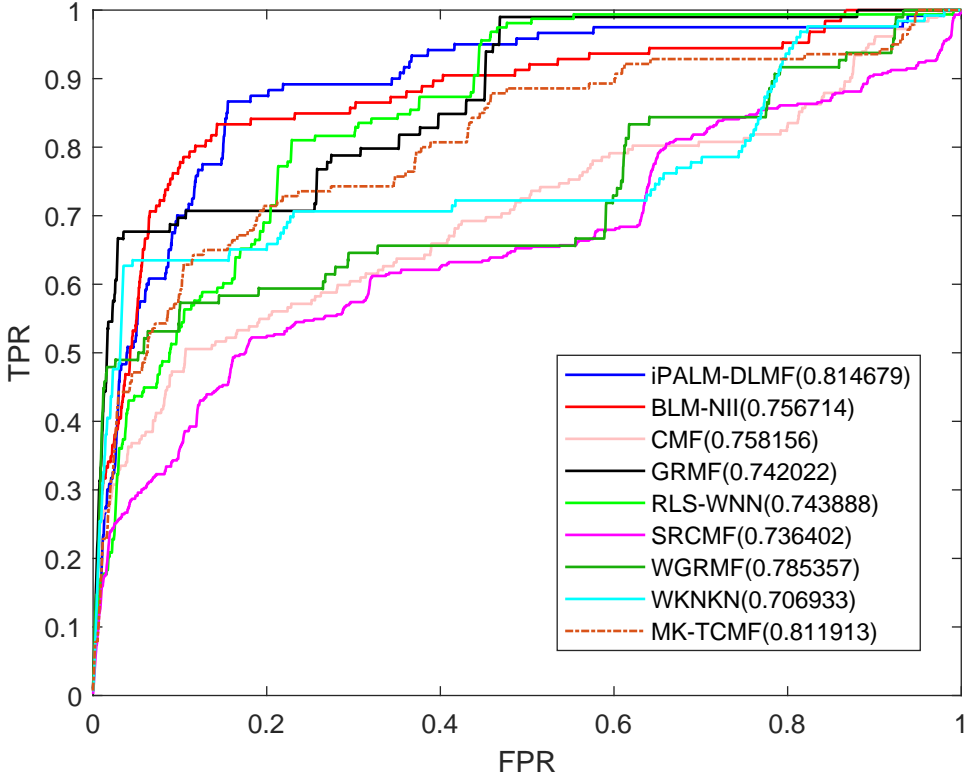

Supplement: Supplementary file 2 — Additional file 2. iPALM-DLMF + appendix. [file 12859_2023_5496_MOESM2_ESM.zip › iPALM-DLMF/rociccvd.pdf]

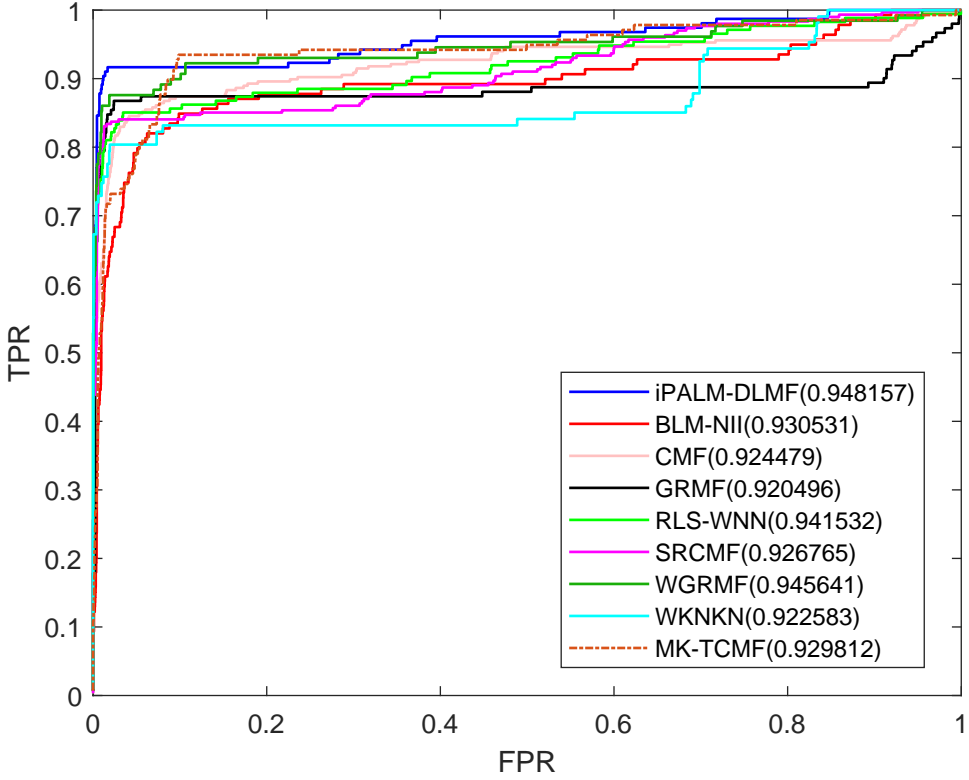

Supplement: Supplementary file 2 — Additional file 2. iPALM-DLMF + appendix. [file 12859_2023_5496_MOESM2_ESM.zip › iPALM-DLMF/rociccvt.pdf]

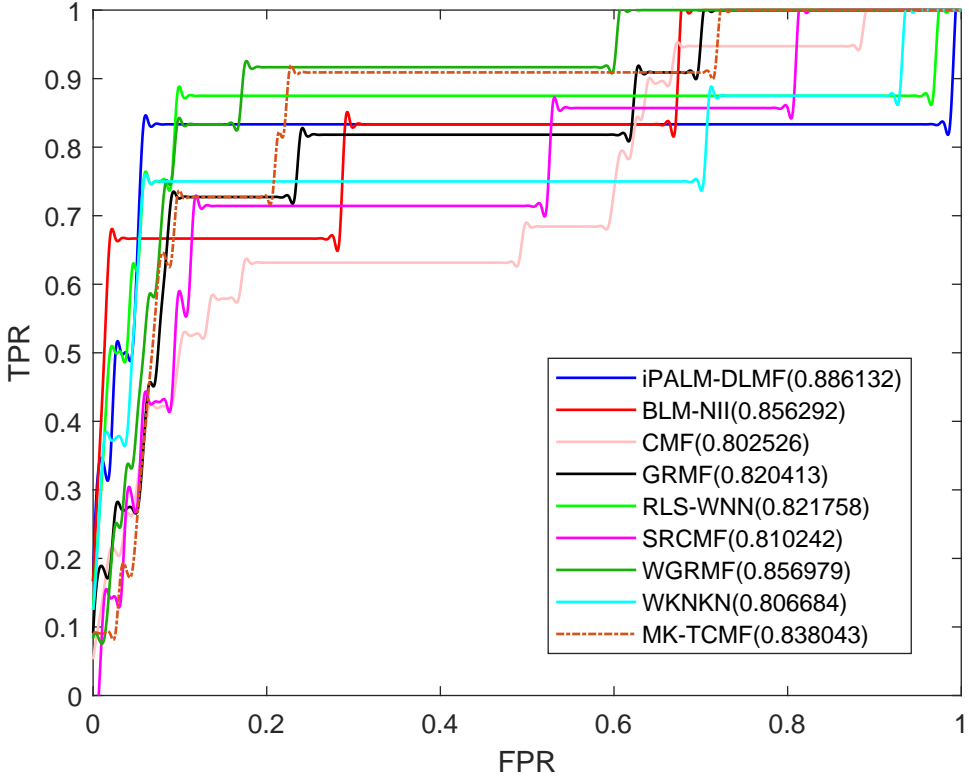

Supplement: Supplementary file 2 — Additional file 2. iPALM-DLMF + appendix. [file 12859_2023_5496_MOESM2_ESM.zip › iPALM-DLMF/rocnrcvd.pdf]

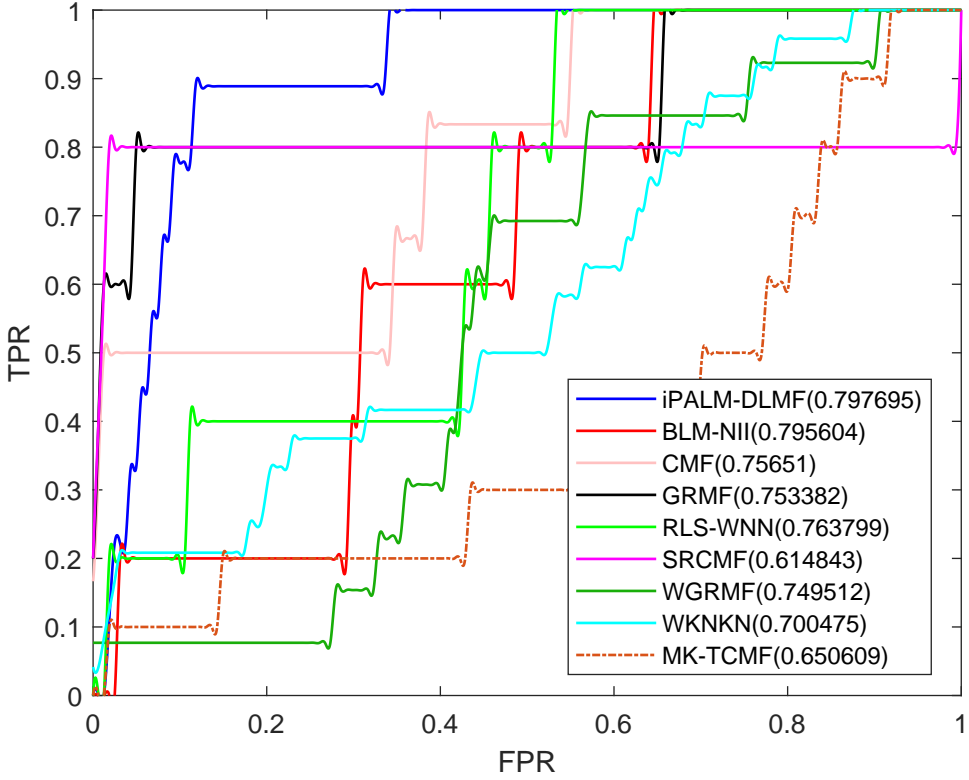

Supplement: Supplementary file 2 — Additional file 2. iPALM-DLMF + appendix. [file 12859_2023_5496_MOESM2_ESM.zip › iPALM-DLMF/rocnrcvt.pdf]
